# Modified Black-Winged Kite Optimization Algorithm with Three-Phase Attacking Strategy and Lévy–Cauchy Migration Behavior to Solve Mathematical Problems

**DOI:** 10.3390/biomimetics10100707

**Published:** 2025-10-17

**Authors:** Yunpeng Ma, Wanting Meng, Ruixue Gu, Xinxin Zhang

**Affiliations:** 1 School of Information Engineering, Tianjin University of Commerce, Beichen, Tianjin 300134, China; 2 School of Mathematics and Statistics, Changsha University of Science and Technology, Changsha 410114, China

**Keywords:** black-winged kite optimization algorithm, three-phase attacking strategy, Lévy–Cauchy operator, CEC testing function

## Abstract

The Black-winged Kite Algorithm (BKA) is a novel heuristic optimization algorithm proposed in 2024, which has demonstrated superior optimization performance on most CEC benchmark functions and several engineering problems. To further enhance its convergence accuracy and solution quality, this paper proposes a Modified Black-winged Kite Algorithm (MBKA). First, a three-phase attacking strategy is designed to replace the original BKA’s attacking mechanism, thereby enhancing population diversity and improving solution quality. Additionally, a Lévy–Cauchy migration strategy is incorporated to achieve a more effective balance between exploration and exploitation. The effectiveness of MBKA is assessed through extensive experiments on 18 classical benchmark functions, the CEC-2017 and CEC-2022 test suites, and two real-world engineering optimization problems. The results indicate that MBKA consistently outperforms the original BKA and several state-of-the-art algorithms in both convergence accuracy and convergence speed across most test cases.

## 1. Introduction

The Black-winged Kite Optimization Algorithm (BKA), initially proposed by Wang et al. in 2024, is a nature-inspired swarm intelligence optimization algorithm that simulates the hunting and migration behaviors of black kites [1]. Owing to its strong global search ability, BKA has been successfully applied to solve a wide range of complex tasks, including high-dimensional function optimization [2,3], constrained engineering design [4,5], parameter tuning [6,7,8,9,10], Internet of Things (IoT) applications [11,12], and path planning [13].

However, like other meta-heuristic algorithms, BKA still exhibits certain inherent limitations. First, in the later stages of iterations, its global exploration ability declines, increasing the likelihood of premature convergence to local optima [14]. Second, its position update mechanism, which is formulated on migratory behavior, suffers from excessive reliance on the global best individual, leading to limited local exploitation efficiency [15]. Third, in the literature [16], a good adaptive adjustment mechanism is lacking in BKA’s attack behavior so that the BKA shows inadequate stability to deal with high-dimensional and multi-modal optimization problems.

In recent years, researchers have proposed various methods to address the limitations of BKA. Li et al. [17] proposed the Reconstructed Black Kite Algorithm (RBKA), which increases the diversity of the initial population through logical mapping. Li et al. [18] developed the DKCBKA algorithm, integrating Osprey optimization with an adaptive index factor to enhance optimization capabilities. Wan et al. [19] proposed the Direction-Assisted Enhanced Black Kite Algorithm (DAEBKA), which incorporates a variable spiral strategy to improve search performance. To address parameter dependency and search instability in BKA, Li et al. [20] introduced an elite guidance mechanism to direct the swarm towards optimal solutions. Li et al. [21] designed the DWBKA algorithm, which combines dynamic position balancing and whale random step strategies to enhance the efficiency of drone trajectory planning in complex orchard environments. Liu et al. [22] proposed the Non-dominated Sorting Black Kite Algorithm (NSBKA), which extends BKA to multi-objective optimization by incorporating non-dominated sorting and crowding distance methods. Xue et al. [23] combined BKA with the Artificial Rabbit Optimization algorithm (ARO), leveraging the advantages of both algorithms to address complex problems effectively. Kalyani Nagarajan [24] applied BKA to the K-anonymization of medical data, achieving a balance between data utility and privacy protection. Yu et al. [25] enhanced BKA by integrating the golden sine search strategy, improving global search capability, reducing local trapping, and accelerating convergence speed. Chen et al. [26] incorporated a greedy strategy into BKA, selectively updating positions to enhance convergence stability and solution robustness. She et al. [27] proposed the MOBK algorithm, which combines weighted Chebyshev scalarization with tent chaotic mapping strategies to improve the overall optimization capability of BKA. Zhang et al. [28] employed an improved BKA to optimize network parameters, enhancing overall model performance. Yang et al. [29] enhanced BKA using lens imaging reverse learning, optimizing the weights and biases of a BP neural network, and applying the improved IBKA-BP model to design an RSSI-based ranging algorithm. Zhao et al. [30] proposed the MBKA algorithm, which strengthens leadership mechanisms in BKA and introduces Morlet wavelet perturbation, thereby improving optimization performance. Zhang et al. [31] introduced a population reconstruction mechanism and developed the REBKA algorithm for applications in machine learning. Shu et al. [32] developed the Multi-Strategy Fusion Enhanced PSO (MSFPSO) algorithm, which incorporates Cauchy variational migration inspired by BKA and enhances both global and local search performance. Li et al. [33] introduced PSBKA, a hybrid algorithm that integrates PSO with BKA.

Despite these numerous improvements, to the best of our knowledge, no research has yet proposed a multi-behavior attacking strategy that integrates iterative phases with a strong perturbation component for dynamically adjusting search intensity. Such a strategy is generally regarded as crucial to balancing global exploration and local exploitation when addressing complex optimization problems.

To address the above limitations, this paper introduces a Modified Black-winged Kite Algorithm (MBKA) that incorporates a three-phase attacking strategy and a Lévy–Cauchy migration behavior into the original BKA framework. The three-stage attack strategy includes predatory behavior, defensive behavior, and competitive behavior. Each behavior employs a distinct individual position update mechanism to enhance population diversity and improve global exploration capability. In addition, three adaptive adjustment parameters (*n*_1_, *n*_2_, and *n*_3_) are designed and applied to three different attack behaviors to enhance population diversity. The Lévy–Cauchy migration behavior enhances the algorithm’s ability to escape local optima. In summary, these improvement strategies enhance the diversity of population individuals, balance exploration and exploitation capabilities, and improve the quality of solutions. Compared to BKA and other well-known optimization algorithms, the proposed MBKA demonstrates superior convergence accuracy on most mathematical test functions, validating the effectiveness of these improvement mechanisms.

The primary contributions of this paper are summarized as follows:

1. Three-phase attacking strategy: A novel three-phase attacking strategy is proposed and integrated into the attacking behavior phase of the original BKA. Simultaneously, three adaptive parameters (*n*_1_, *n*_2_, and *n*_3_) are designed for predatory, defensive, and competitive attacking behavior, respectively. The proposed strategy increases individual diversity and balances exploration and exploitation capabilities.
2. Lévy–Cauchy migration behavior: A Lévy–Cauchy migration behavior is designed to improve BKA’s global search capabilities and facilitate escape from local optima, thereby enhancing solution quality.
3. Comprehensive experimental validation: The proposed MBKA is evaluated on extensive mathematical problems, including 18 benchmark test functions, 29 CEC-2017 [34] test functions, 12 CEC-2022 [35] test functions, and two practical engineering design problems.

The remainder of this paper is organized as follows: Section 2 introduces the BKA, including its initialization, attacking, and migration behaviors, and elaborates on the MBKA, covering the three-phase attacking strategy, Lévy–Cauchy migration behavior, pseudo-code, and complexity analysis. Section 3 presents experimental validations of MBKA, including performance tests on benchmark functions, CEC-2017 functions, CEC- 2022 functions, and engineering optimization problems (compression spring and gear train design problems). Section 4 discusses the research’s alignment with prior work, theoretical and practical implications, and MBKA’s limitations. Section 5 draws conclusions and explores future research directions.

## 2. Materials and Methods

### 2.1. Black-Winged Kite Optimization Algorithm

#### 2.1.1. Initialization Phase

In BKA, the population is initialized with a set of random solutions to represent the location of each black-winged kite (*BK*), and the exact location is represented by the following matrix:

(1) $$BK=\left[\begin{array}{c} BK_{1,1}\;\;\;BK_{1,2}\;\;\;\ldots \;\;\;BK_{1,D}\\ BK_{2,1}\;\;\;BK_{2,2}\;\;\ldots \;\;\;BK_{2,D}\\ \;\;\;\vdots \;\;\;\;\;\;\;\;\;\;\;\vdots \;\;\;\;\;\;\;\ddots \;\;\;\;\;\;\vdots \;\\ BK_{N,1}\;\;\;BK_{N,1}\;\;\ldots \;\;\;BK_{N,D} \end{array}\right]\;$$

Here, N represents the number of potential solutions, i.e., the population size, D is the dimension size of the solution, and ${BK}_{ij}$ is the jth dimension of the ith black-winged kite. At each population initialization, we randomly assign the position of each black-winged kite according to Equation (2).

(2) $${BK}_{i}=BK_{lb}+rand\;*\;(BK_{ub}-BK_{lb})$$

where i is an integer between 1−N, $BK_{lb}$ and $BK_{ub}$ are the lower and upper bounds of the solution, respectively, and rand is a random number between [0, 1].

#### 2.1.2. Attacking Behavior

The black-winged kite adjusts the angles of its wings and tail to suit the conditions during flight, hovering silently to observe its prey and then swooping quickly to attack. In this phase, the black-winged kite shows different attack behaviors for global exploration. The mathematical model of the attack behavior is summarized as follows:

(3) $${BK}_{t+1}^{i,j}=\left\{\begin{array}{c} {BK}_{t}^{i,j}+n\times (1+\sin(r))\times {BK}_{t}^{i,j}\;\;p<r\\ {BK}_{t}^{i,j}+n\times (2r-1)\times {BK}_{t}^{i,j}\;\;\;\;\;\;p\geq r \end{array}\right.$$

(4) $$n=0.05\times e^{-2\times (\frac{t}{T}{)}^{2}}$$

In Equations (3) and (4), ${BK}_{t}^{i,j}$ and ${BK}_{t+1}^{i,j}$ denote the position of the ith black-winged kite in the jth dimension at the tth and $\left(t+1\right)$th iteration steps, respectively, where r is a random number ranging between [0,1], p is a constant value, T represents the maximum iteration number, and t is the current iteration number.

#### 2.1.3. Migration Behavior

A hypothesis regarding the migration behavior in the algorithm is proposed by drawing on the characteristics of avian migration: For the maximum optimization problem, the black-winged kite with the highest fitness value is regarded as a leader. If the fitness of the leader is inferior to that of a randomly chosen individual, the leader abdicates its leadership and joins the migratory population. On the contrary, if the fitness value of the leader is superior to that of all individuals, the individuals’ behavior will be guided by the leader. This strategy can ensure the success of the migration by dynamically selecting outstanding leaders. The mathematical model of black-winged kite migratory behavior is described in Equation (5):

(5) $${BK}_{t+1}^{i,j}=\left\{\begin{array}{c} {BK}_{t}^{i,j}+C(0,1)\times ({BK}_{t}^{i,j}-L_{t}^{j})\;\;\;\;\;\;\;\;\;F_{i}<F_{ri}\\ {BK}_{t}^{i,j}+C(0,1)\times (L_{t}^{j}-m\times {BK}_{t}^{i,j})\;\;\;F_{i}\geq F_{ri} \end{array}\right.$$

(6) m=2×sin(r+π/2)

(7) $$f(x,\delta ,\mu )=\frac{1}{\pi }\frac{1}{x^{2}+1},\;\;-\infty <x<+\infty$$

In Equation (5), $F_{i}$ represents the current position in the jth dimension obtained by any black-winged kite in the t iteration, and $F_{ri}$ represents the fitness value of the random position in the jth dimension obtained from any black-winged kites in the *t* iteration. C(0, 1) is the Cauchy mutation, which is defined in the Equation (7), where δ=1 and u=0.

### 2.2. Modified Black-Winged Kite Algorithm

In this section, a Modified Black-Winged Kite Algorithm (MBKA) is proposed and described in detail. Firstly, the attacking behavior in BKA is reformulated as a three-phase strategy consisting of predatory, defensive, and competitive behaviors. This strategy enhances population diversity and balances exploration with exploitation. Additionally, the Lévy flight coefficient is introduced in the migration behavior model to enhance the ability to escape local optima. The MBKA is described in detail as follows.

#### 2.2.1. Three-Phase Attacking Strategy

In the original BKA, it has two ways to update an individual’s position in the attacking behavior phase, which may lead to the loss of population diversity and insufficient global search ability. Therefore, an attacking operator epl is introduced in the attacking behavior phase, and, by constructing the relationship between three parameters (p,r,epl), the attacking behavior is divided into three categories: predatory attacking behavior, defensive attacking behavior, and competitive attacking behavior. Simultaneously, the original parameter n in the Formula (4) is expanded into the three distinct forms $n_{1}$, $n_{2}$, and $n_{3}$, as shown in the Formula (8). These three parameters may improve the comprehensiveness and flexibility of the proposed algorithm.

(8) $$\left\{\begin{array}{c} n_{1}=0.05\times e^{-2\times (\frac{t}{T}{)}^{2}}\\ n_{2}=\frac{e^{\frac{t}{T}}}{1-0.9\times e^{\frac{t}{T}}}\\ n_{3}=0.05\times e^{-\frac{t}{T}}\times (1+\frac{t}{T}) \end{array}\right.$$

In regards to predatory attacking behavior, when p<r−epl, the black-winged kite, as a predator, employs a series of strategies to approach and capture its prey. The mathematical model of the predatory attacking behavior is shown in the Formula (9).

(9) $${BK}_{t+1}^{i,j}={BK}_{t}^{i,j}+n_{1}\times (1+\sin(r))\times {BK}_{t}^{i,j}$$

Seen from the Formula (9), the sin(r)∈[−1, 1], so the (1+sin(r))∈[0, 2]. During the iterative process, the adaptive varying coefficient $n_{1}\times (1+\sin(r))$ could have two effects:

When $\sin\left(r\right)\approx 1$, the adaptive varying coefficient approaches $2n_{1}$, resulting in a relatively large positive step size that propels the individual to accelerate towards the optimal region.

When $\sin\left(r\right)\approx -1$, the coefficient approaches 0, leading to a tiny step size that prevents overshooting the optimal solution.

This dynamic scaling mechanism enables the algorithm to conduct global search with large step sizes in the initial stages of iteration and automatically reduces the step sizes in the later stages of iteration to achieve precise convergence.

**Figure 1:**
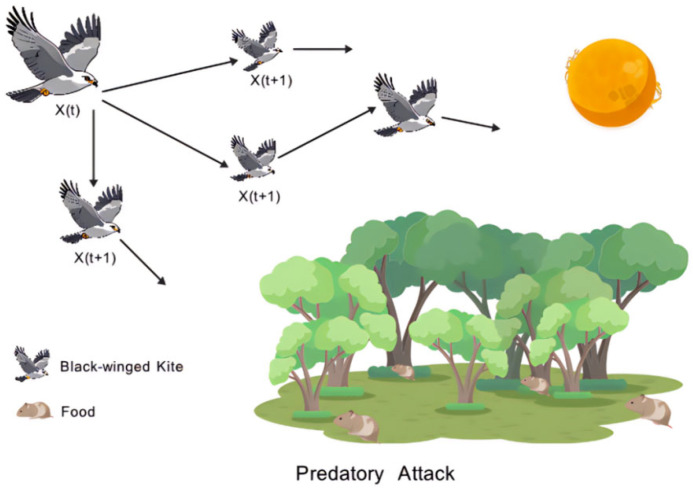
Schematic diagram of predatory attack state.

As shown in Figure 1, it presents a schematic diagram of the attack state of the black-winged kite when it acts as a predator.

In regards to defensive attacking behavior, when p>r−epl, the black-winged kite perceives a threat and employs defensive aggression to protect itself from harm caused by predators or competitors. The mathematical model of the defensive attacking behavior is shown in the Formula (10).

(10) $${BK}_{t+1}^{i,j}={BK}_{t}^{i,j}+n_{2}\times (2r-1)\times {BK}_{t}^{i,j}$$

Seen from the Formula (10), r∈[0, 1], so 2r−1∈[−1, 1]. And the adaptive parameter $n_{2}\times (2r-1)$ could dynamically adjust the perturbation intensity during the iterative process. When this coefficient acts on the current position, the following effects will occur:

When 2r−1≈1, a positive perturbation generates, causing the individual’s position to shift in the current direction.

When 2r−1≈−1, a negative perturbation generates, causing the individual’s position to shift in the opposite direction.

This stochastic perturbation mechanism is particularly crucial when the algorithm traps in local optima. By conducting small-scale explorations around the current position, the algorithm gains the opportunity to escape from local extreme points and discover better search directions. In particular, the parameter $n_{2}$ gradually decreases in the later stages of iteration to ensure the stability of the algorithm as it approaches the global optimal solution.

**Figure 2:**
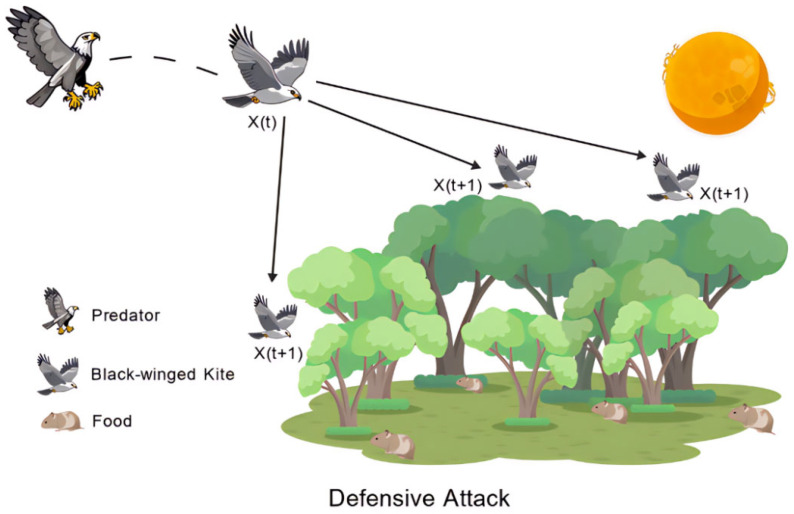
Schematic diagram of defensive attack state.

Figure 2 depicts a schematic illustration of the defensive attack posture of the black-winged kite when defending against natural predators, visually highlighting its defensive stances and offensive strategies.

In regards to competitive attacking behavior, when p=r−epl, the black-winged kites engage in resource competition, which is characterized by competitive aggression. The mathematical model of the competitive attacking behavior is shown in the Formula (11).

(11) $${BK}_{t+1}^{i,j}={BK}_{t}^{i,j}+n_{3}\times (\sin(r)+\cos(r))\times {BK}_{t}^{i,j}$$

Seen from the Formula (11), the range of $Sin\left(r\right)+Cos\left(r\right)=\sqrt{2}sin(r+\frac{\Pi }{4})$ is $\left[-\sqrt{2},\;\;\sqrt{2}\right]$. The adaptive parameter $n_{3}\times \left(Sin\left(r\right)+Cos\left(r\right)\right)\;$could balance the algorithm’s exploration and exploitation capabilities. This dynamic adjustment mechanism enables the algorithm to avoid premature convergence to local optima during resource competition while maintaining population diversity as it approaches the global optimal solution.

In regards to global exploration, when $\sin\left(r+\frac{\Pi }{4}\right)\approx \pm 1$, the coefficient approaches $\pm \sqrt{2}n_{3}$, generating a relatively large step size that propels the individual to conduct extensive searches across the solution space. This facilitates the discovery of new potential optimal regions.

In regards to local exploitation, when $\sin\left(r+\frac{\Pi }{4}\right)\approx 0$, the coefficient approaches 0, resulting in a tiny step size. This prompts the individual to perform fine-grained searches around the current optimal solution, facilitating the utilization of existing information to improve the precision of the solution.

**Figure 3:**
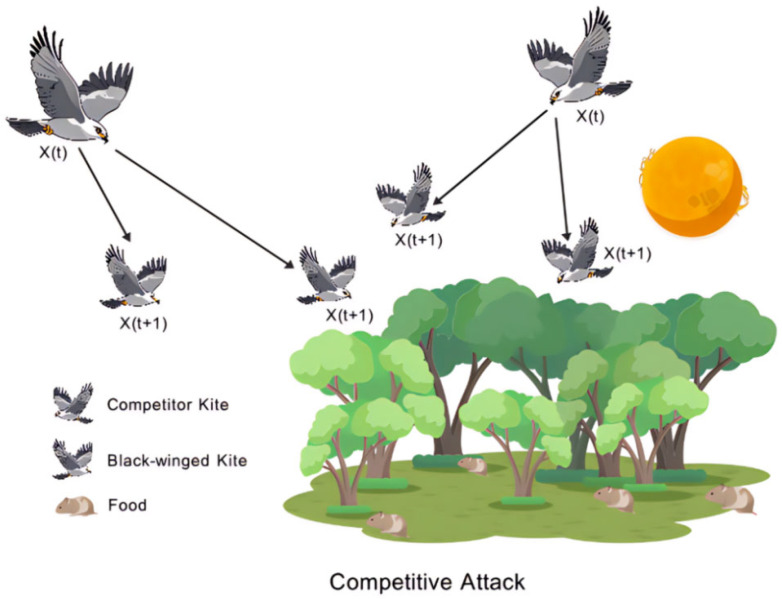
Schematic diagram of competitive attack state.

As shown in Figure 3, the competitive attacking state of the black-winged kite in competitive situations is intuitively presented, allowing for the observation of specific attack behavior patterns when dealing with competition.

**Figure 4:**
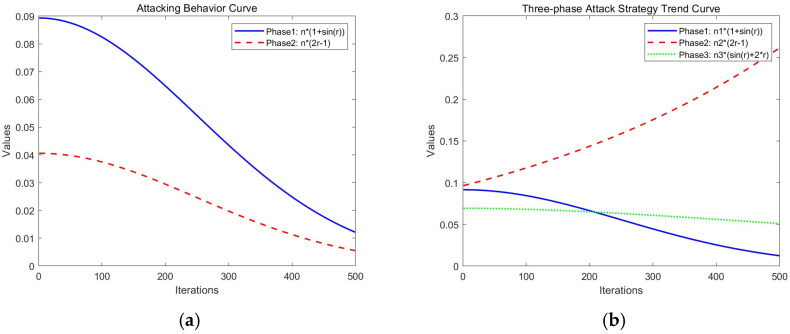
Comparison diagram of attack behavior variation curves.

The variation curves of attack behaviors in the original BKA and the three-phase attack behavior changes in the MBKA are shown in Figure 4.

As shown in Figure 4, the left graph depicts the variation curve of attacking behaviors in BKA, while the right graph illustrates the three-phase attacking behavior change curve of MBKA. In BKA, as the number of iterations increases, the intensity of the Phase 1 attack (i.e., the magnitude of position update) gradually decreases, indicating that the algorithm is gradually converging; although the parameter value of Phase 2 also shows a downward trend, its overall variation pattern differs from that of Phase 1, and it can provide a certain perturbation capability in the early stage of iteration. In the right graph, the three-phase attacking strategies (predatory attacking, defensive attacking, and competitive attacking) exhibit different variation trends and complement each other during the iteration process. As the number of iterations increases, the defensive attack gradually strengthens, enabling the full utilization of local search capabilities and accelerating convergence as the optimal solution is approached. Simultaneously, the predatory attacking gradually decreases in strength, allowing the algorithm to focus its search in the later stages. The competitive attacking remains largely stable throughout the entire process, acting as a perturbation balancing mechanism that effectively maintains population diversity.

#### 2.2.2. Lévy–Cauchy Migration Behavior

In this subsection, a kind of Lévy–Cauchy migration behavior is designed. The Lévy– flight is an outstanding model of a random walk. Its step size distribution follows a specific power law, a unique property that enables it to integrate local exploration and global search efficiently.

Unlike simple random walks, the step sizes in Lévy flight are not uniformly distributed. It exhibits a pattern of interspersed large and short step sizes. Large step sizes enable the algorithm to rapidly explore distant regions within the search space, which is crucial for the algorithm to avoid getting trapped in local optimal solutions. Short step sizes, on the other hand, allow for a more refined search in the vicinity of potentially advantageous regions, thereby enhancing the efficiency of mining and utilization of potential high-quality solutions.

Mathematically, the probability density function of the step sizes in the Lévy flight is shown in Equation (12):

(12) $$P_{\alpha ,\beta }(x)=\left\{\begin{array}{c} \frac{1}{\sqrt{2\pi }}x^{-\frac{3}{2}}\exp\left(-\frac{1}{2x}\right),\;\;\;x\geq 0\\ 0,\;\;\;\;\;\;\;\;\;\;\;\;\;\;\;\;\;\;\;\;\;\;\;\;\;\;\;\;\;\;\;\;\;\;\;x\leq 0 \end{array}\right.$$

**Figure 5:**
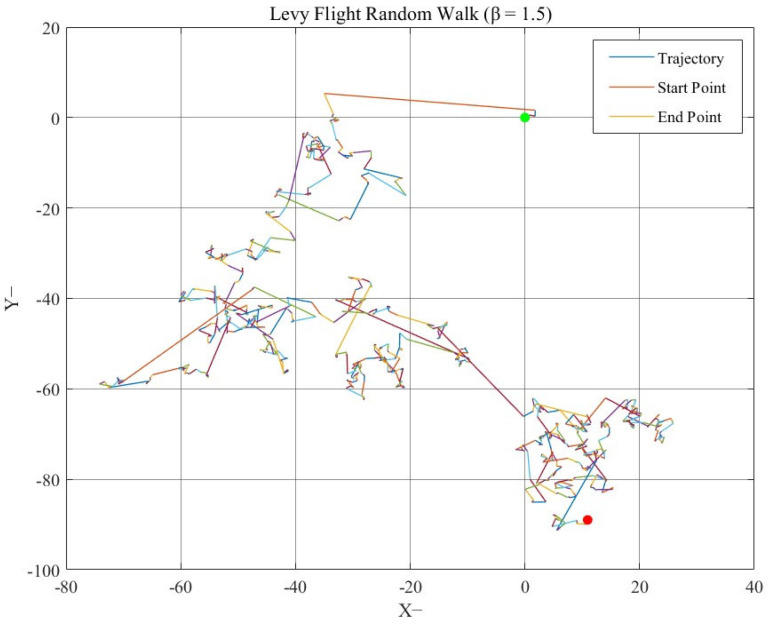
Trajectory diagram of Lévy flight.

To intuitively understand the unique walking mechanism of the Lévy flight, its trajectory diagram is shown in Figure 5.

In the Figure 5, the green dot denotes the end point of the Lévy flight random walk, while the red dot indicates a key feature point in the trajectory.

In this migration behavior, when $F_{i}<F_{ri}$, the f Lévy light is used to replace the original Cauchy value, resulting in a new position update formula with enhanced perturbation capabilities. The Lévy flight could help individual populations escape from local optimal solutions and increase population diversity. The mathematical model of Lévy–Cauchy migration behavior is provided in the Formula (13).

(13) $${BK}_{t+1}^{i,j}=\left\{\begin{array}{c} {BK}_{t}^{i,j}+Levy\times {BK}_{t}^{i,j}\;\;\;\;\;\;\;\;\;\;\;\;\;\;\;\;F_{i}<F_{ri}\\ {BK}_{t}^{i,j}+C\left(0,1\right)\times \left(L_{t}^{j}-m\times {BK}_{t}^{i,j}\right)\;\;F_{i}\geq F_{ri} \end{array}\right.$$

### 2.3. Pseudo-Code of MBKA

According to the above analysis, the pseudo-code of the MBKA is given in Algorithm 1.
**Algorithm 1: Pseudo-code of MBKA**Input: T: the maximum iterations D: variable dimensions of the problem to be addressed p: the constant value in attack behavior Establish an objective function F(x),where variable $X=(x_{1},x_{2},x_{3},...,x_{d})$. Initialize a population of N black-winged kites using a random initialization method. Output: $x_{best}$, $f_{g}$ 1: while the maximum iterations M is not met do 2: Rank the fitness values, identify the current best individual $f_{g}$ and worst individuals $f_{w}$, and simultaneously calculate the average fitness of the population $f_{m}$. 3: for i=1:N 4: Update the position of the black-winged kites population through enhanced attack by Equations (8)–(11) and migration behaviors by Equations (12) and (13) 5: end for 3: Get the current new location; 4: If the current new location is better than before, update it; 5: t=t+1 6: edge detection 7: end while 8: return $x_{best}$, $f_{g}$.

### 2.4. Complexity Analysis

In this subsection, the time complexity and space complexity are used to evaluate the proposed MBKA. The time complexity is related to the execution speed of the algorithm, and its impact is significant when dealing with large-scale datasets. The space complexity measures the memory usage, which determines the algorithm’s performance in memory-constrained environments. Evaluating these two aspects can ensure that the MBKA has good operability and scalability under various conditions.

The time complexity of both BKA and MBKA is O(T × N × D), where T denotes the number of iterations, N is the population size, and D represents the problem dimension. The initialization phase has a complexity of O(N × D), while the core iterative phase processes D dimensions for each of the N individuals in each iteration, resulting in a per-iteration complexity of O(N × D). After T iterations, the total complexity accumulates to O(T × N × D). Although MBKA incorporates more sophisticated strategies, its computations remain dimension-wise operations, thus maintaining the same asymptotic time complexity.

The space complexity of both BKA and MBKA is O(N × D). Both algorithms require storing a position matrix for N D-dimensional individuals, which constitutes the primary space overhead. Although MBKA introduces additional parameters (such as attack operators and Lévy flight parameters), these parameters are either fixed in number or independent of D, thus not affecting the asymptotic order of the overall space complexity. Consequently, both algorithms exhibit the same memory footprint, linearly dependent on the population size N and problem dimension D.

## 3. Results

### 3.1. Experimental Evaluation

This section systematically evaluates the performance of the MBKA in addressing complex global optimization problems. A unified parameter configuration framework is adopted for the experiment, where the population size is 40 and the maximum iteration number is 500. The computational environment consists of a Windows 11 Professional workstation equipped with an AMD Ryzen 7 8845H processor (3.80 GHz), 16.0 GB of DDR5 RAM, and an 8 GB discrete graphics card (supporting multi-GPU parallel computing). The MATLAB R2024b scientific computing platform is used for simulating the algorithm.

The algorithm parameter settings adhere to the principle of consistency: MBKA inherits the core parameter system of the original BKA, while the comparative algorithms (including MPA, ALO, GWO, etc.) adopt the default parameter configurations recommended in the literature. The complete parameter list for all algorithms is detailed in Table 1. To ensure the statistical significance of the experimental results, each test is independently run 30 times. The performance differences among the algorithms are comprehensively evaluated in terms of convergence accuracy, stability, convergence speed, and global search capability by calculating the mean and standard deviation and plotting convergence curves.

In this paper, the core role of the parameter epl is to divide the three phases of the attacking behavior in the MBKA algorithm. By regulating the threshold relationship between epl and the population state parameters p and r, different phases of attacking strategies are triggered. After preliminary experimental verification, the basic parameter value is finally determined as epl=0.1. Meanwhile, to analyze the parameter sensitivity, two additional comparison schemes are set: epl=0.2 (corresponding to the algorithm MBKA1) and epl=0.3 (corresponding to the algorithm MBKA2). All epl values are constants (not changing with the number of iterations) to ensure the stability of the division of the three-phase behavior and avoid the interference of dynamic parameters on the core logic of the algorithm.

In terms of statistical analysis, the study employed a two-stage verification strategy. First, the Wilcoxon rank-sum test was performed at a significance level of α=0.05 to verify whether the performance differences between MBKA and each comparative algorithm were statistically significant. Subsequently, the Friedman test was used to rank all compared algorithms comprehensively. As non-parametric statistical methods, the above tests do not require assuming a specific data distribution type, making them particularly suitable for analyzing multi-index performance differences in complex optimization scenarios and ensuring the robustness and objectivity of experimental conclusions.

### 3.2. Performance Testing on Benchmark Functions

The test suite consists of 18 functions: F1–F9 are uni-modal functions with global optima for evaluating MBKA’s optimization performance, while F10–F18 are multi-modal functions with multiple local extrema for assessing exploratory capability. Detailed information is in Table 2 and Table 3.

**Figure 6:**
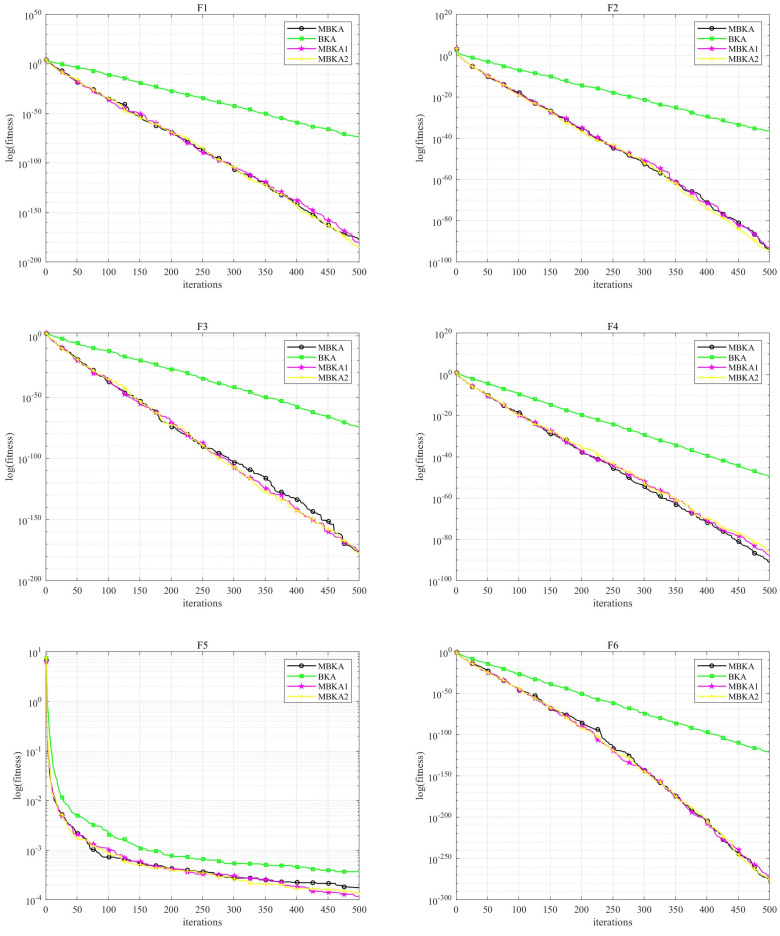

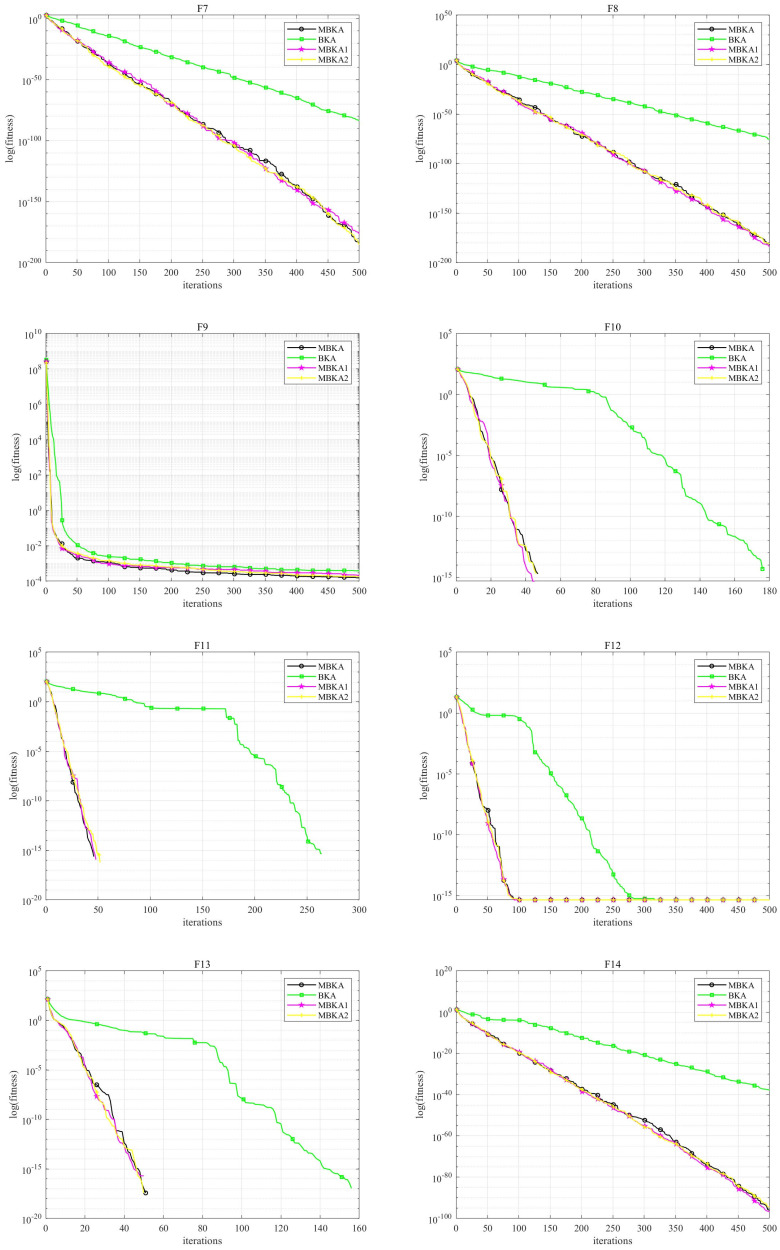

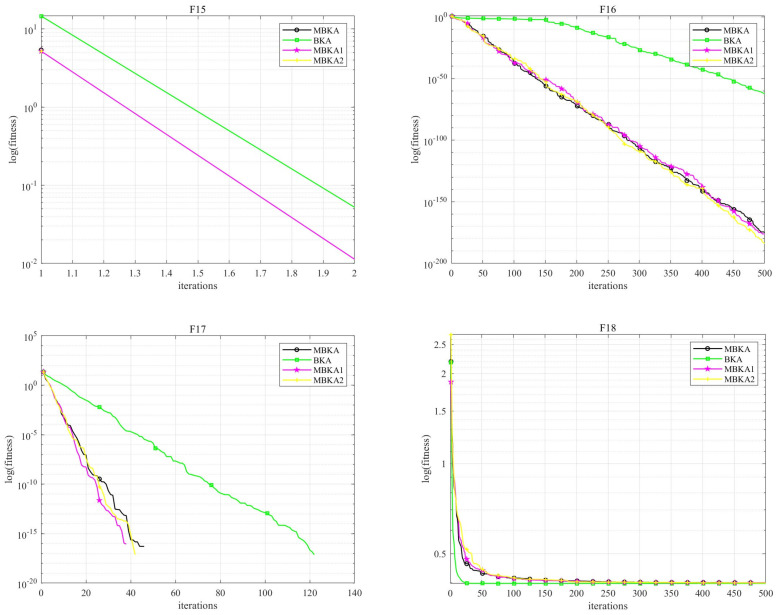
Convergence curves for F1–F18 benchmark functions of MBKA and its variants.

To evaluate the parameter sensitivity and time performance of the MBKA algorithm, MBKA is compared with BKA, MBKA1, and MBKA2. The convergence curves are shown in Figure 6, and the relevant results are presented in Table 4.

Based on the experimental verification of the F1–F18 benchmark test functions (refer to Figure 6 and Table 4), the determination of the parameter epl= 0.1 is derived from a comparative analysis between the MBKA and its variant algorithms, namely MBKA1 (epl= 0.2 and MBKA2 epl= 0.3). In terms of the average fitness index, taking F1 as an example, the average value of MBKA is 1.2047 × 10^−94^, which outperforms MBKA1 (6.7213 × 10^−94^) and MBKA2 (2.2697 × 10^−95^). In terms of stability, MBKA exhibits a standard deviation of 0 in functions such as F3 and F7, demonstrating superior operational consistency. Comprehensively considering optimization accuracy and stability, epl= 0.1 is ultimately determined as the optimal value.

In terms of runtime, there is a noticeable difference between MBKA and the comparison algorithm BKA. For F1, the average runtime per execution of MBKA is 0.9139 s, while that of BKA is 0.0254 s. In F3, MBKA has an average runtime of 0.9875 s per execution, compared to 0.0638 s for BKA. Although MBKA requires slightly longer runtime, its advantages in average fitness (e.g., in F3, MBKA reaches 3.549 × 10^−178^ while BKA is 3.067 × 10^−75^) and stability across most of the F1–F18 functions fully confirm that its comprehensive optimization performance is more suitable for addressing complex optimization tasks.

**Figure 7:**
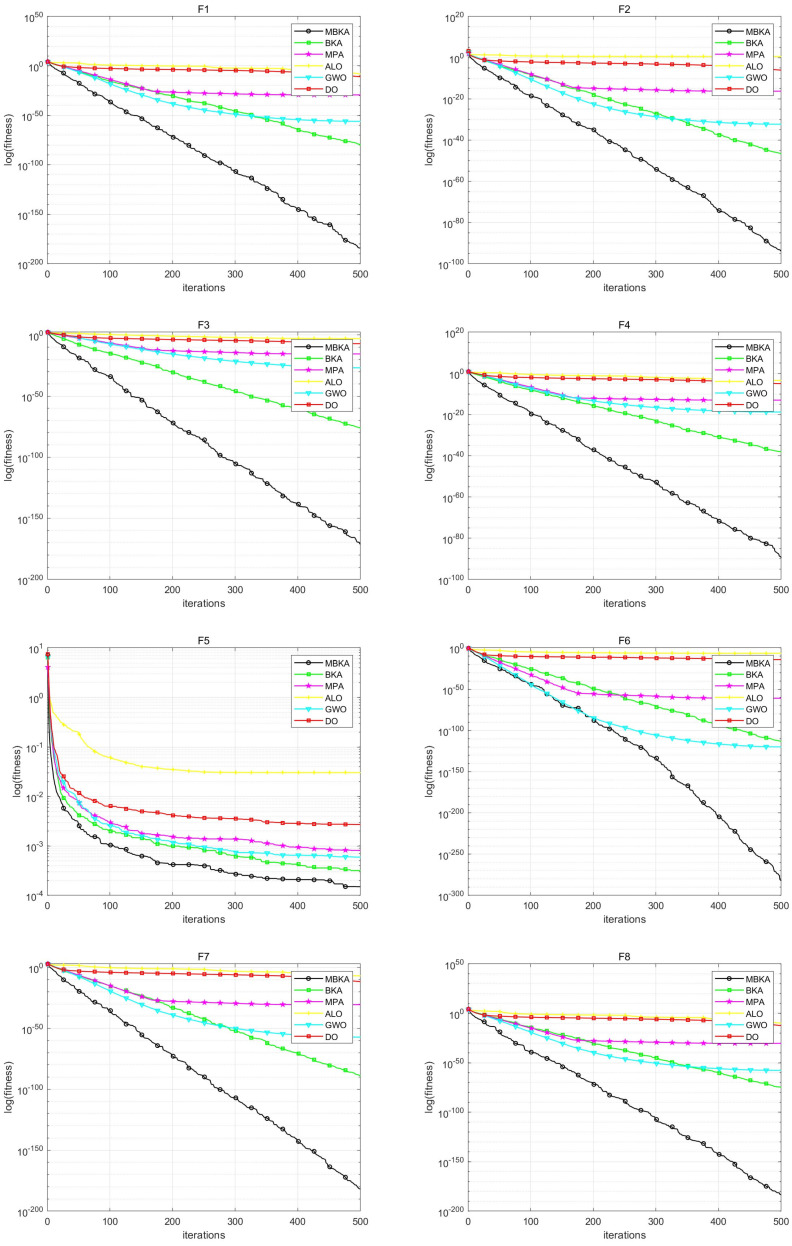

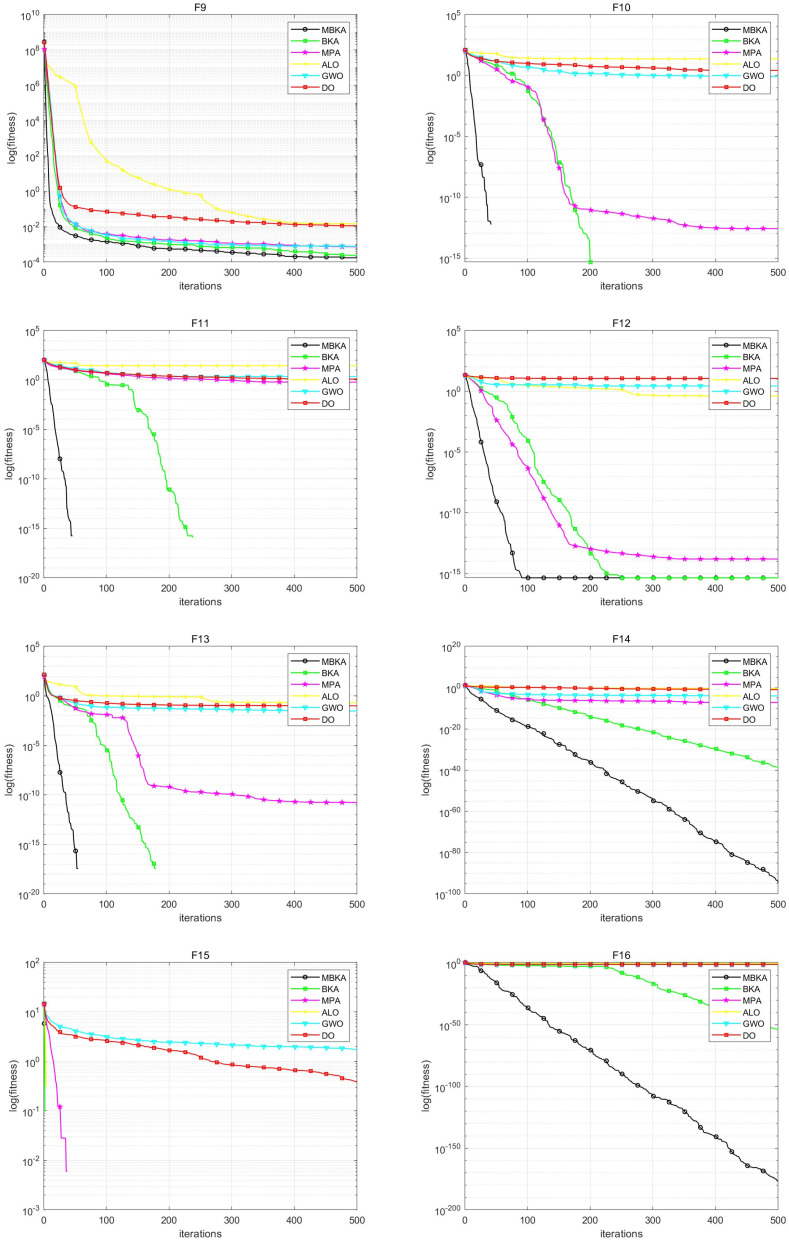

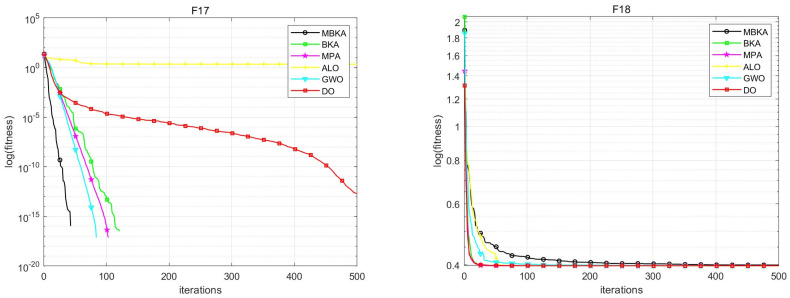
F1–F18 benchmark function comparison images.

To assess the performance of the MBKA algorithm, we conducted a comprehensive comparative analysis involving MBKA, BKA, MPA, ALO, GWO, and DO algorithms. The convergence curves for each algorithm are illustrated in Figure 7, and the detailed simulation results are presented in Table 5, offering valuable insights for further optimization and performance evaluation.

The results presented in Table 5 indicate that the MBKA algorithm generally outperforms the other algorithms in terms of average performance across most functions. While it shows slightly lower performance on functions F9 and F18, MBKA achieves theoretical optimal values for the remaining functions, particularly F10, F11, F15, and F17, highlighting its remarkable capabilities. Additionally, Figure 7 demonstrates MBKA’s significant advantage in convergence speed, consistently outperforming other algorithms in this aspect. This suggests that MBKA not only achieves superior final results but also exhibits high convergence efficiency, underscoring its effectiveness and practicality for solving optimization problems.

### 3.3. Performance Testing on CEC-2017

In this study, to evaluate the performance of the proposed MBKA algorithm, 29 10-dimensional functions from the CEC-2017 benchmark test function set were selected for testing. Since the CEC2017-F2 function shows poor stability in high-dimensional cases, it was not included in the test scope. The CEC-2017 benchmark function set primarily covers four types of functions: first, uni-modal functions (F1–F3); second, simple multi-modal functions (CEC2017-F4–CEC2017-F10); third, hybrid functions (CEC2017-F11–CEC2017-F20); and fourth, synthesis functions (CEC2017-F21–CEC2017-F30). Compared with other test functions, the CEC-2017 benchmark function set has richer functional characteristics and can more comprehensively and accurately reflect the algorithm’s performance.

**Figure 8:**
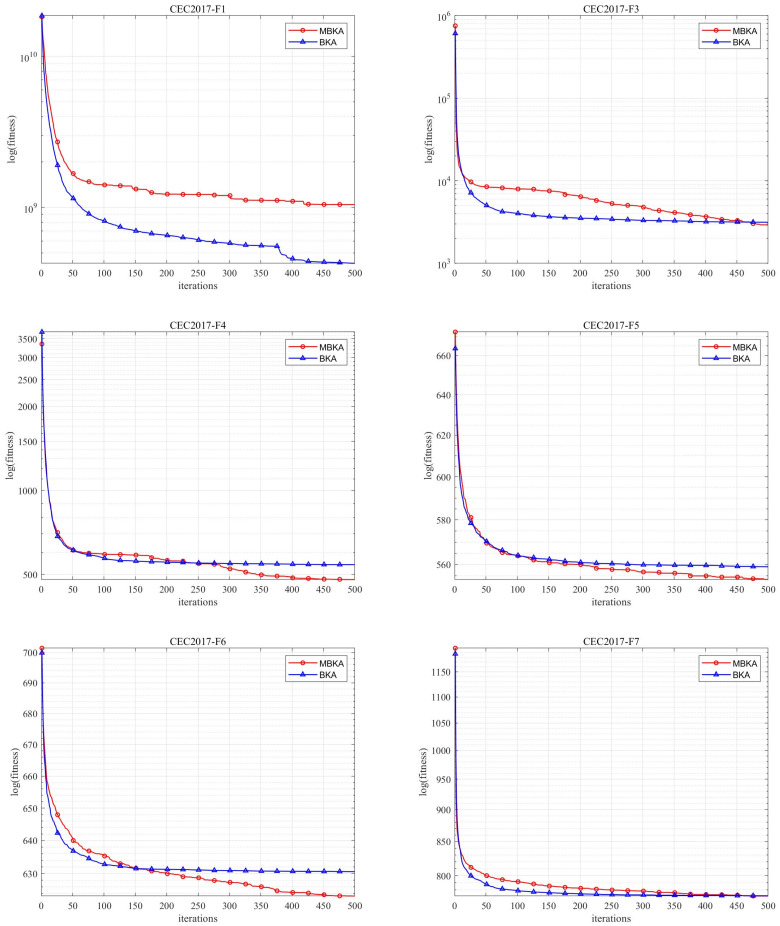

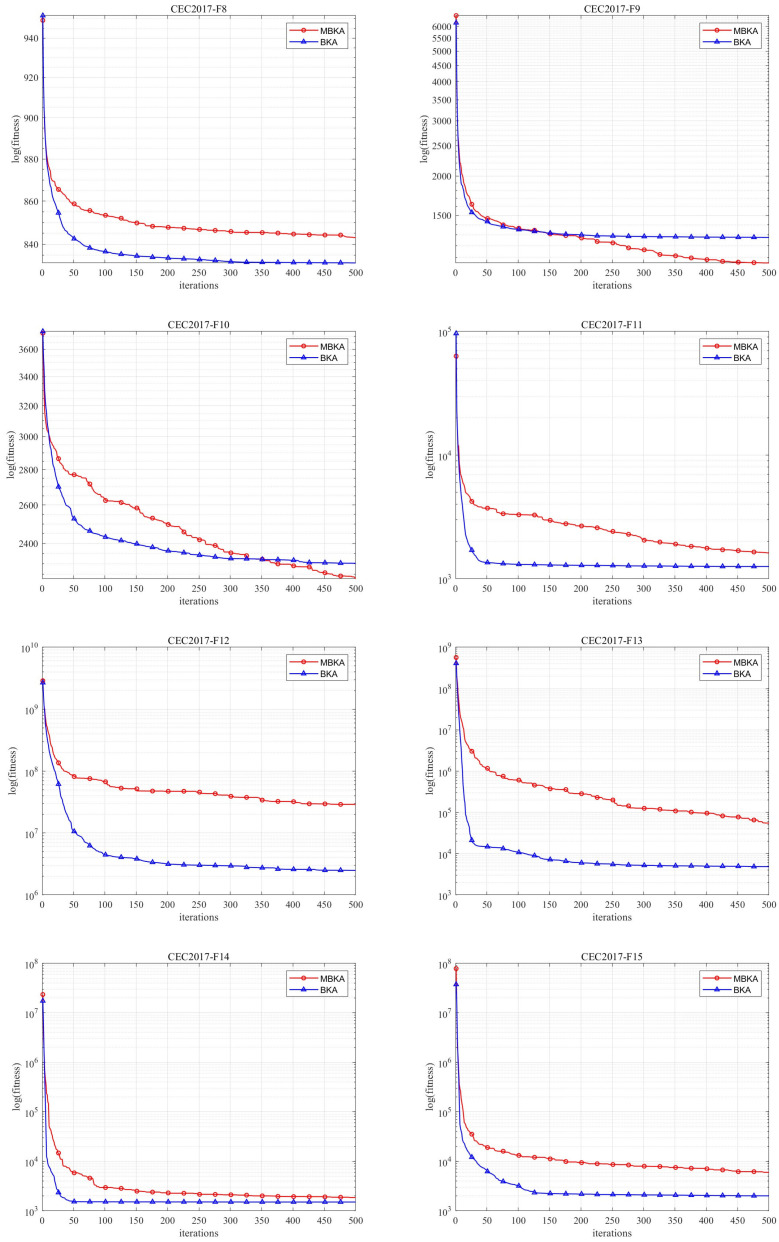

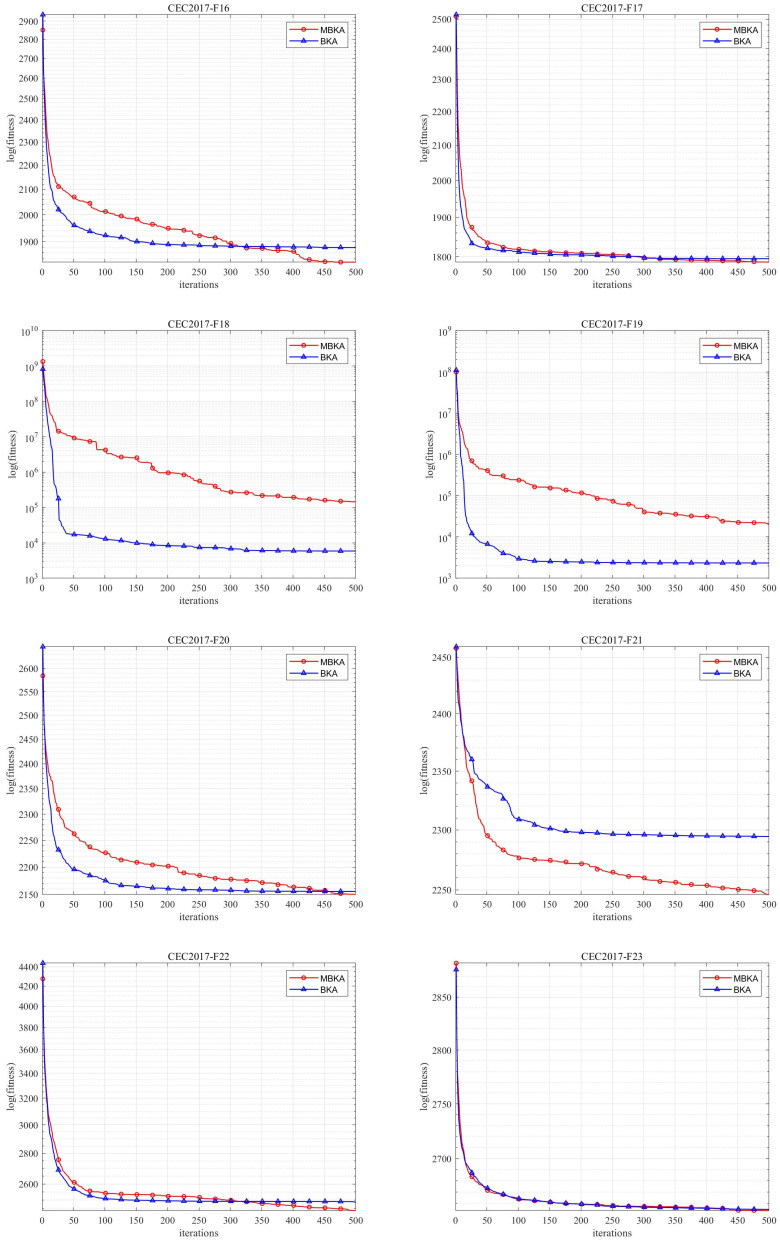

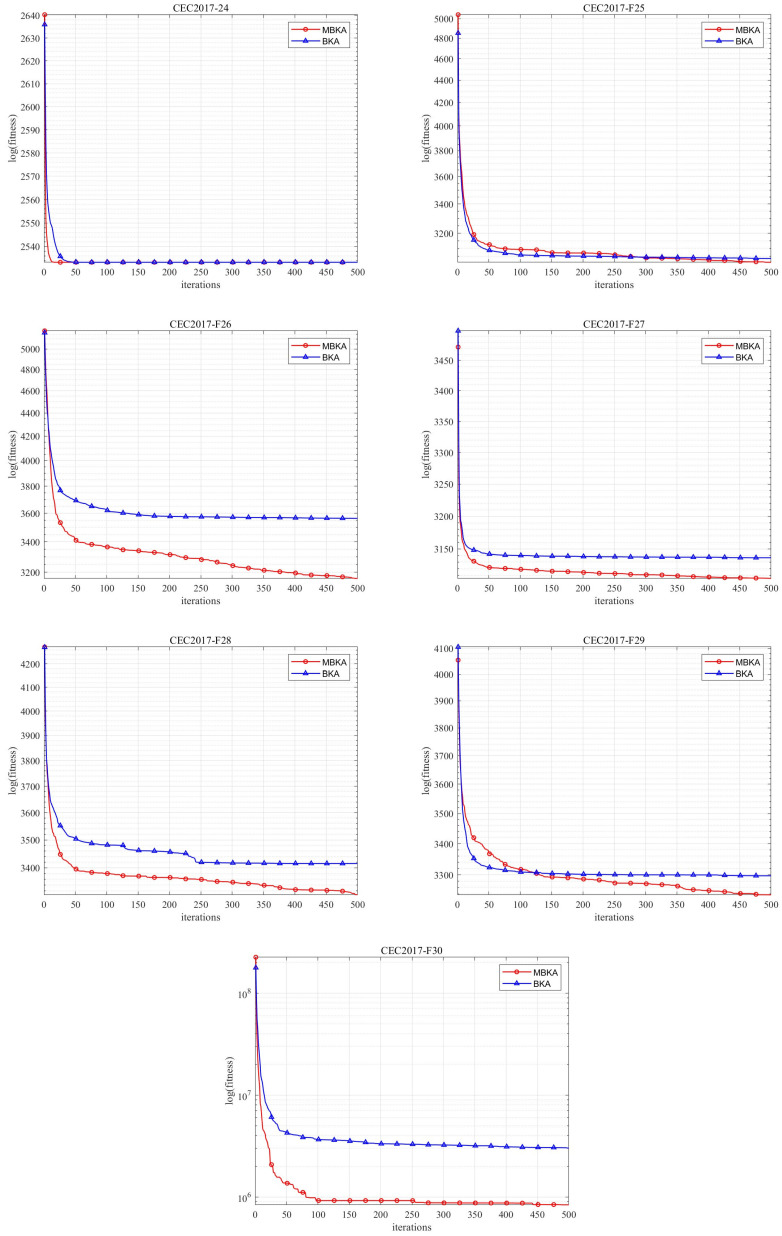
CEC-2017 benchmark function comparison images.

Figure 8 presents the convergence curves of MBKA and BKA on the CEC-2017 benchmark functions; Table 6 lists the corresponding experimental result data.

Judging from the convergence curves and numerical results, the MBKA has extremely prominent advantages over the BKA. Taking the CEC2017-F1 function as an example, on the convergence curve, the MBKA drops rapidly in the initial stage of iteration. After about 100 iterations, the error value has decreased to a relatively low level, while the error value of the BKA during the same period is significantly higher, indicating that the MBKA converges faster. In terms of numerical results, the minimum value of the MBKA for the CEC2017-F6 function, 6.12 × 10^2^, is smaller than that of the BKA, 6.16 × 10^2^. In multiple functions including F11, the MBKA also shows higher solution accuracy. Regarding stability, the standard deviation of the MBKA for the CEC2017-F6 function, 6.33 is smaller than that of the BKA 7.29. The same characteristic can be seen in functions such as CEC2017-F23, indicating that the results of multiple runs of the MBKA are more stable. Moreover, the *p*-values between the MBKA and the BKA for most functions are extremely small. For example, the *p*-value of the CEC2017-F11 function reaches 3.88 × 10^−6^, indicating a statistically significant difference. In conclusion, the MBKA far surpasses the BKA in terms of convergence, solution accuracy, stability, and other comprehensive performance aspects. It fully demonstrates its advantages when dealing with various functions in CEC-2017, and has high application value in practical engineering problems.

### 3.4. Performance Testing on CEC-2022

The CEC-2022 test suite is the most recent and widely used testing collection currently. Its functions are mainly categorized into four types: uni-modal function (CEC2022-F1), basic functions (CEC2022-F2–CEC2022-F5), hybrid functions (CEC2022-F6–CEC2022-F8), and combined functions (CEC2022-F9–CEC2022-F12). These test functions are renowned for their complex structures, which pose significant challenges during the solving process.

**Figure 9:**
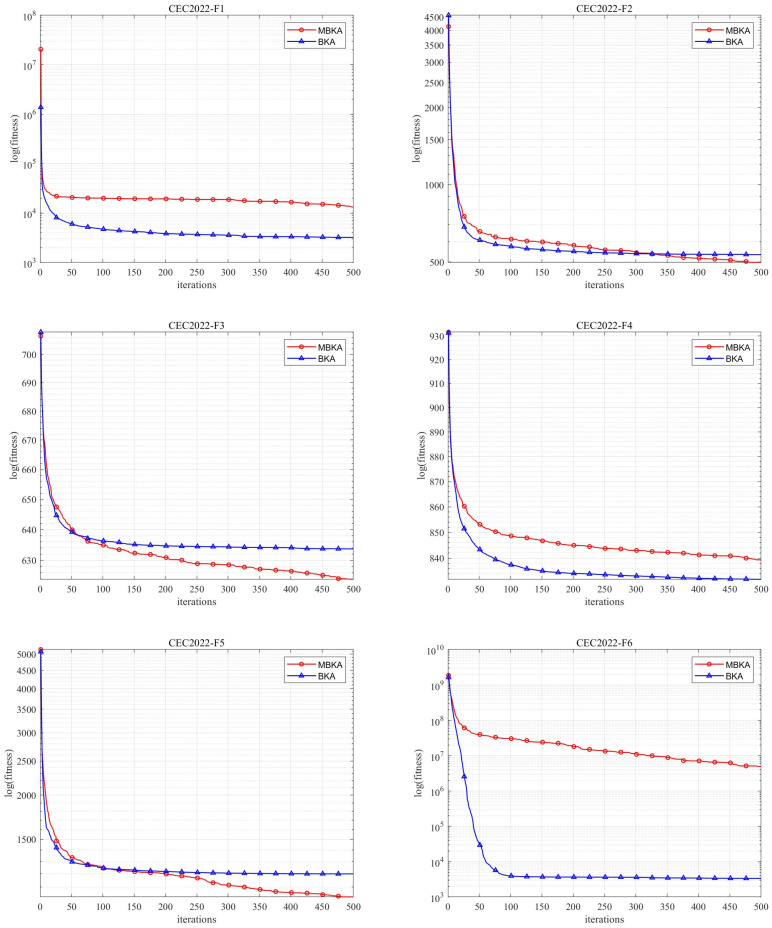

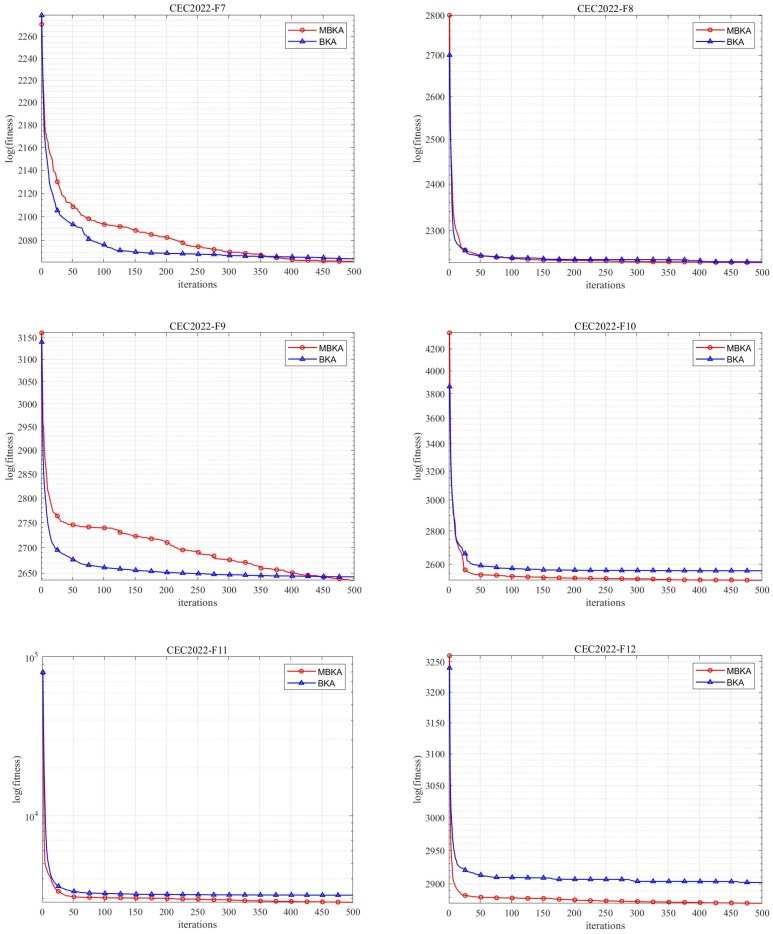
CEC-2022 benchmark function comparison images.

In this study, a performance comparison was conducted between BKA and MBKA on the CEC-2022 test suite. Each algorithm was run independently 30 times, and the results in ten dimensions are presented here. Figure 9 shows the convergence curves of MBKA and BKA on the CEC-2022 benchmark test functions, while Table 7 lists the corresponding experimental data results.

The MBKA has significant advantages over the BKA. In terms of convergence curves, MBKA converges faster in most benchmark functions (such as CEC2022-F1, CEC2022-F3, CEC2022-F6, etc.). From the numerical results, the minimum values of multiple functions (such as CEC2022-F1, CEC2022-F4, CEC2022-F5, etc.) show that MBKA has a higher solution accuracy. The standard deviation data indicate that MBKA has better stability in some functions (such as CEC2022-F2). Moreover, for most functions, the *p*-values of the comparison between MBKA and BKA are small, indicating a significant difference. In summary, MBKA outperforms BKA in terms of convergence, solution accuracy, stability, and other comprehensive performance aspects, and it has more advantages in solving practical engineering problems.

### 3.5. Engineering Optimization Problem

Addressing practical engineering challenges is a key objective in the research of swarm intelligence algorithms [40]. While test function results offer preliminary insights into the algorithm’s performance, they do not comprehensively reflect its effectiveness in real-world applications. To evaluate the efficacy of the Modified Black-winged Kite Algorithm (MBKA) in engineering contexts, this study investigates two representative real-world problems: the compression spring design problem and the gear train design problem.

#### 3.5.1. Compression Spring Design Problem

**Figure 10:**
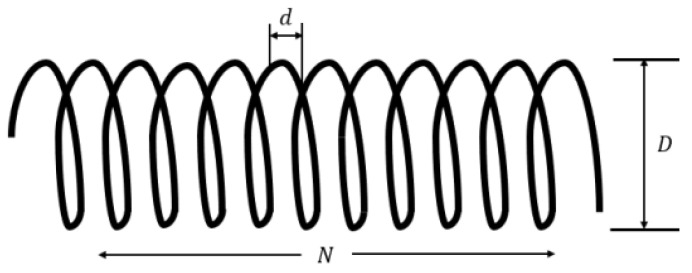
The image of compression spring.

The goal of designing compression springs is to minimize the weight of the spring while meeting minimum deflection, vibration frequency, and shear stress constraints. As shown in Figure 10 and Table 8, the problem involves three successive design variables: the spring coil diameter d, the spring coil diameter D, and the number of coils wound N.

$$\mathrm{Consider}\mathrm{variable}:\;H=[h_{1},h_{2},h_{3}]=[d,D,N].$$

$$\mathrm{Minimize}:\;f\left(H\right)=\left(h_{3}+2\right)\times h_{2}h_{1}^{2}.$$

$$\mathrm{Subject}\mathrm{to}:\;l_{1}(H)=-\frac{h_{2}^{3}h_{3}}{71,785h_{1}^{4}}+1\leq 0,$$

$${\;l}_{2}\left(H\right)=\frac{4h_{2}^{2}-h_{1}h_{2}}{12,566\left(h_{1}^{3}h_{2}-h_{1}^{4}\right)}+\frac{1}{5108h_{1}^{2}}-1\leq 0,l_{3}(H)=-\frac{140.45h_{1}}{h_{2}^{2}h_{3}}+1\leq 0,$$

$$l_{4}(H)=-1+\frac{h_{1}+h_{2}}{1.5}\leq 0.$$

Table 8 presents the statistical results for each algorithm applied to the compression spring design problem, with optimal results highlighted in bold. The data indicate that the MBKA algorithm consistently outperforms the original BKA algorithm, achieving superior optimal values compared to all other algorithms. Moreover, when compared with other algorithms involved in this experiment like WOA, ALO, and DO, MBKA also achieves superior optimal values. These findings suggest that MBKA provides valuable insights and demonstrates excellent performance for tackling the compression spring design problem.

#### 3.5.2. Gear Train Design Problem

**Figure 11:**
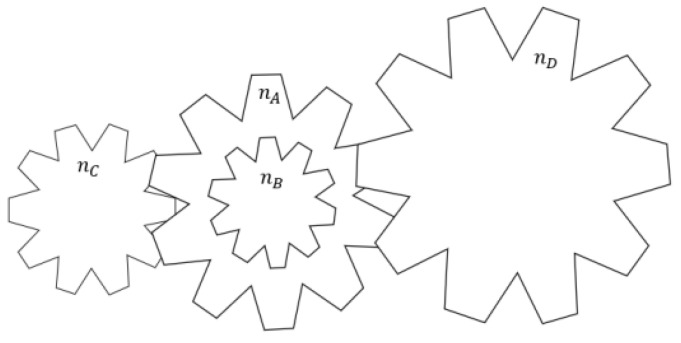
The image of gear train.

The gear train design problem is a typical unconstrained discrete design problem in mechanical engineering where the main objective is to minimize the gear ratio, which is defined as the ratio of the angular velocity of the output shaft to the angular velocity of the input shaft. As shown in Figure 11 and Table 9,$n_{A},n_{B},n_{C},n_{D}$ represents the coefficients of tour different gears. The structure of the wheel train design problem is shown schematically:

$$\mathrm{Consider}\mathrm{variable}:\;H=[h_{1},h_{2},h_{3},h_{4}]=[n_{A},n_{B},n_{C},n_{D}].$$

$$\mathrm{Minimize}:\;f(H)={\left(\frac{1}{6.931}-\frac{h_{3}h_{2}}{h_{1}h_{4}}\right)}^{2}.$$

$$\mathrm{Variable}\mathrm{range}:\;12\leq x_{i}\leq 60,i=1,2,3,4$$

Shown in Table 8, although the MBKA algorithm shows slightly lower variance compared to the DO algorithm, it achieves superior optimal values and consistently outperforms all other algorithms in terms of average performance across multiple runs. This suggests that the MBKA algorithm not only exhibits high convergence but also demonstrates robust performance. When applied to the optimization of four variables in the gear train design problem, the MBKA algorithm identified the optimal gear configuration. These results indicate that the MBKA algorithm possesses a significant advantage in solving the gear train design problem.

## 4. Discussion

### 4.1. Alignment with Prior Research and Hypothesis Validation

The Modified Black-winged Kite Algorithm (MBKA) overcomes the main drawbacks of the original Black-winged Kite Algorithm (BKA), such as weakened exploration in the later stage, excessive reliance on global optimal individuals for position updates, and inflexible attack behavior. Its three-phase attacking strategy, along with the attack operator epl and adaptive parameters ($n_{1}$,$n_{2}$,$n_{3}$), allows for coordinated adjustment of predatory, defensive, and competitive behaviors. This design confirms the hypotheses that the three-phase strategy increases population diversity and that Lévy–Cauchy migration enhances the ability to escape local optima, which is reflected in the better performance of benchmark functions and multi-modal functions after the table modifications.

### 4.2. Theoretical and Practical Implications

Theoretically, MBKA’s “behavioral modularization” approach, which decomposes bio-inspired behaviors into adaptive sub-mechanisms, provides a scalable framework for other nature-inspired algorithms to balance exploration and exploitation. Practically, with the revised tables showing more accurate data, MBKA performs well in compression spring and gear train design tasks, outperforming BKA and other algorithms in optimal values, thus meeting the needs of high-precision mechanical optimization in fields like aerospace and manufacturing.

### 4.3. Limitations

MBKA still has some limitations. Parameters such as epl and those in Lévy flight are set empirically and may lack adaptability in ultra-high-dimensional or dynamic scenarios. It focuses on single-objective optimization and cannot handle multi-criteria engineering problems. Also, its computational efficiency decreases in large-population settings due to the three-phase attacking strategy, and this can be better explained with the more accurate data from the revised tables.

## 5. Conclusions

A Modified Black-winged Kite Algorithm (MBKA) is proposed in this paper by introducing a three-phase attacking strategy and Lévy–Cauchy migration behavior to improve the BKA’s performance. An attacking operator epl and three adaptive parameters ($n_{1}$, $n_{2}$, and $n_{3}$) are designed to enrich the location update pattern of population individuals and improve adaptability for avoiding trapping into local optima. With the revised tables providing more accurate and complete data, the experimental results, obtained by testing on 18 benchmark functions, as well as CEC-2017/CEC-2022 functions and two practical engineering problems, reveal that the proposed MBKA has better convergence accuracy and solution quality than the original BKA on most testing functions. Therefore, the proposed improved mechanisms are obviously effective.

In the future, we will continue to conduct in-depth research on MBKA. We will integrate the causal reasoning model and the embodied intelligence concept into MBKA to further improve its performance. Specifically, causal reasoning can be used for the real-time adjustment of parameters like epl by learning the causal relationship between function types and optimal parameter values from historical iteration data. Embodied intelligence can be employed to simulate dynamic constraints in real environments. In addition, we will integrate multi-objective optimization mechanisms, such as non-dominated sorting referenced from NSBKA, into MBKA to solve more complex practical optimization problems that involve multiple conflicting objectives.

## Figures and Tables

**Table 1:** The parameters of each algorithm.

Algorithm	Parameters
BKA	p=0.9
MPA [36]	$p=0.9,\;{FAD}_{s}=0.2$
ALO [37]	−−
GWO [38]	α=2→0, linearly decrease
DO [39]	$a=\frac{1}{T^{2}-2T+1},\;b=-2a,\;c=1-a-b,$ $k=1-rand()\times (c+at^{2}+b\times t)$

**Table 2:** Uni-modal test functions.

Function	Range	Optima
$F_{1}(x)=\sum_{i=1}^{n}x_{i}^{2}$	[−100, 100]^n^	0
$F_{2}(x)=\sum_{i=1}^{D}[y_{i}^{2}-10\cos(2\pi y_{i})+10],\;y_{i}=\left\{x_{i},\left|x_{i}<0.5\right|\right\}$	[−10, 10]^n^	0
$F_{3}(x)=\sum_{i=1}^{D}(\sum_{j=1}^{i}x_{j}{)}^{2}$	[−100, 100]^n^	0
$F_{4}(x)=\max_{i}\left\{\left|x_{i}\right|,1\leq x_{i}\leq D\right\}$	[−10, 10]^n^	0
$F_{5}(x)=\sum_{i=1}^{D}D\times x_{i}^{2}+rand(0,1)$	[−1.28, 1.28]^n^	0
$F_{6}(x)=\sum_{i=1}^{D}{\left|x_{i}\right|}^{\left(i+1\right)}$	[−1, 1]^n^	0
$F_{7}(x)=\sum_{i=1}^{D}D\times x_{i}^{2}$	[−10, 10]^n^	0
$F_{8}(x)=\sum_{i=1}^{D}x_{i}^{2}+{\left(\sum_{i=1}^{D}0.5ix_{i}\right)}^{2}+{\left(\sum_{i=1}^{D}0.5ix_{i}\right)}^{4}$	[−5, 10]^n^	0
$F_{9}(x)=\sum_{i=1}^{D}ix_{i}^{4}$	[−1.28, 1.28]^n^	0

**Table 3:** Multi-modal test functions.

Function	Range	Optima
$F_{10}(x)=\sum_{i=1}^{D}\left[x_{i}^{2}-10\cos(2\pi x_{i})+10\right]$	[−5.12, 5.12]^n^	0
$F_{11}(x)=\sum_{i=1}^{D}\left[y_{i}^{2}-10\cos(2\pi y_{i})+10\right],\;y_{i}=\left\{\begin{array}{c} x_{i},\left|x_{i}\right|<0.5\\ round(2x_{i})/2,\left|x_{i}\right|>0.5 \end{array}\right.$	[−5.12, 5.12]^n^	0
$F_{12}(x)=-20\exp(-0.2\sqrt{\frac{1}{D}\sum_{i=1}^{D}x_{i}^{2}})+10+e$	[−50, 50]^n^	0
$F_{13}(x)=-\frac{1}{4000}\sum_{i=1}^{D}x_{i}^{2}-\prod_{i=1}^{D}\cos(\frac{x_{i}}{\sqrt{i}})+1$	[−600, 600]^n^	0
$F_{14}(x)=\sum_{i=1}^{D}\left|x_{i}\times \sin(x_{i})+0.1x_{i}\right|$	[−10, 10]^n^	0
$F_{15}(x)=\sum_{i=1}^{D\sum }\left(\sum_{k=0}^{k_{max}\sum }\left[a^{k}\cos(2\pi b^{k}(x_{i+0.5}))\right]\right)$ $D\cdot \sum_{k=0}^{k_{max}\sum }[a^{k}\cos\geq (2\pi b^{k}\cdot 0.5)],a=0.5,b=3,k_{max}$	[−1, 1]^n^	0
$F_{16}(x)=1-\cos\left(2\pi \sqrt{\sum_{i=1}^{D}x_{i}^{2}}\right)+0.1\sqrt{\sum_{i=1}^{D}x_{i}^{2}}$	[−100, 100]^n^	0
$F_{17}(x)=\sum_{i=1}^{D-1}\left[x_{i}^{2}+2x_{i+1}^{2}-0.3\cos(3\pi x_{i})-0.4\cos(4\pi x_{i+1})+0.7\right]$	[−10, 10]^n^	0
$F_{18}(x)=0.5+\pi \left({\left(\mathrm{Sin}\sum_{i=1}^{D}x_{i}^{2}\right)}^{2}-0.5\right)\times {\left(1+1.001(\sum_{i=1}^{D}x_{i}^{2})\right)}^{-2}$	[−100, 100]^n^	0

**Table 4:** Simulation results of MBKA and its variants on F1–F18 benchmark functions.

	Index	MBKA	BKA	MBKA1	MBKA2
$F_{1}$	Mean	1.349 × 10^−177^	2.345 × 10^−74^	4.742 × 10^−181^	8.254 × 10^−186^
	SD	0	1.048 × 10^−73^	0	0
	runtime	0.9268	0.0255	0.9221	0.9133
$F_{2}$	Mean	1.205 × 10^−94^	1.697 × 10^−37^	6.721 × 10^−94^	2.269 × 10^−95^
	SD	6.473 × 10^−94^	9.284 × 10^−37^	3.662 × 10^−93^	1.233 × 10^−94^
	runtime	0.9139	0.0254	0.9078	0.9111
$F_{3}$	Mean	3.549 × 10^−178^	3.067 × 10^−75^	1.642 × 10^−177^	2.149 × 10^−178^
	SD	0	1.357 × 10^−74^	0	0
	runtime	0.9875	0.0638	0.9832	0.9796
$F_{4}$	Mean	6.198 × 10^−92^	6.399 × 10^−50^	1.244 × 10^−88^	2.337 × 10^−87^
	SD	2.800 × 10^−91^	2.573 × 10^−49^	4.957 × 10^−88^	1.279 × 10^−86^
	runtime	0.9155	0.0243	0.9119	0.9103
$F_{5}$	Mean	1.757 × 10^−4^	3.679 × 10^−4^	1.167 × 10^−4^	1.264 × 10^−4^
	SD	1.074 × 10^−4^	2.987 × 10^−4^	8.388 × 10^−5^	8.811 × 10^−5^
	runtime	1.2435	0.0757	1.2570	1.2567
$F_{6}$	Mean	1.323 × 10^−278^	5.382 × 10^−122^	1.532 × 10^−274^	2.659 × 10^−277^
	SD	0	2.010 × 10^−121^	0	0
	runtime	1.0066	0.0446	1.0059	1.0052
$F_{7}$	Mean	3.667 × 10^−184^	2.881 × 10^−84^	4.284 × 10^−177^	1.448 × 10^−185^
	SD	0	×10^−^	0	0
	runtime	0.9877	0.0241	0.9790	0.9864
$F_{8}$	Mean	4.772 × 10^−183^	6.402 × 10^−76^	8.967 × 10^−185^	1.509 × 10^−181^
	SD	0	3.507 × 10^−75^	0	0
	runtime	1.0377	0.0608	1.0378	1.0381
$F_{9}$	Mean	1.542 × 10^−4^	3.835 × 10^−4^	2.146 × 10^−4^	1.854 × 10^−4^
	SD	1.132 × 10^−4^	3.085 × 10^−4^	1.618 × 10^−4^	1.489 × 10^−4^
	runtime	1.4517	0.0832	1.4617	1.4541
$F_{10}$	Mean	0	0	0	0
	SD	0	0	0	0
	runtime	1.3702	0.0385	1.3686	1.3780
$F_{11}$	Mean	0	0	0	0
	SD	0	0	0	0
	runtime	1.3520	0.0392	1.3514	1.3490
$F_{12}$	Mean	4.441 × 10^−16^	4.441 × 10^−16^	4.441 × 10^−16^	4.441 × 10^−16^
	SD	0	0	0	0
	runtime	1.0762	0.0318	1.0903	1.0939
$F_{13}$	Mean	0	0	0	0
	SD	0	0	0	0
	runtime	1.0188	0.0426	1.0144	1.0148
$F_{14}$	Mean	6.941 × 10^−97^	1.872 × 10^−38^	1.327 × 10^−97^	2.695 × 10^−95^
	SD	3.379 × 10^−96^	1.021 × 10^−37^	5.999 × 10^−97^	1.022 × 10^−94^
	runtime	0.9932	0.0284	0.9849	0.9917
$F_{15}$	Mean	0	0	0	0
	SD	0	0	0	0
	runtime	2.8541	1.6083	2.8462	2.8825
$F_{16}$	Mean	6.774 × 10^−176^	5.147 × 10^−63^	5.168 × 10^−177^	1.322 × 10^−184^
	SD	0	×10^−62^	0	0
	runtime	0.9556	0.0281	0.9487	0.9508
$F_{17}$	Mean	0	0	0	0
	SD	0	0	0	0
	runtime	0.9264	0.0264	0.9201	0.9235
$F_{18}$	Mean	3.999 × 10^−1^	3.979 × 10^−1^	3.997 × 10^−1^	3.992 × 10^−1^
	SD	1.999 × 10^−3^	0	2.911 × 10^−3^	1.793 × 10^−3^
	runtime	0.7129	0.0253	0.7155	0.7142

**Table 5:** Simulation results of MBKA and comparative algorithm on F1–F18.

	Index	MBKA	BKA	MPA	ALO	GWO	DO
$F_{1}$	Mean	7.89 × 10^−185^	3.74 × 10^−81^	5.59 × 10^−30^	8.04 × 10^−9^	9.94 × 10^−57^	1.46 × 10^−11^
	SD	0	2.05 × 10^−80^	1.21 × 10^−29^	4.45 × 10^−9^	3.65 × 10^−56^	2.27 × 10^−11^
	*p*	NA	1.73 × 10^−6^	1.73 × 10^−6^	1.73 × 10^−6^	1.73 × 10^−6^	1.73 × 10^−6^
	Fri	1	2	4	6	3	5
$F_{2}$	Mean	1.46 × 10^−94^	2.24 × 10^−47^	5.69 × 10^−17^	3.76	5.23 × 10^−33^	1.00 × 10^−6^
	SD	7.63 × 10^−94^	1.17 × 10^−46^	5.41 × 10^−17^	9.94	7.67 × 10^−33^	7.34 × 10^−7^
	*p*	NA	1.73 × 10^−6^	1.73 × 10^−6^	1.73 × 10^−6^	1.73 × 10^−6^	1.73 × 10^−6^
	Fri	1	2	4	6	3	5
$F_{3}$	Mean	7.45 × 10^−172^	7.64 × 10^−77^	2.89 × 10^−16^	1.00 × 10^−3^	1.43 × 10^−27^	6.85 × 10^−8^
	SD	0	4.19 × 10^−76^	4.25 × 10^−16^	3.29 × 10^−3^	6.24 × 10^−27^	1.66 × 10^−7^
	*p*	NA	1.73 × 10^−6^	1.73 × 10^−6^	1.73 × 10^−6^	1.73 × 10^−6^	1.73 × 10^−6^
	Fri	1	2	4	6	3	5
$F_{4}$	Mean	2.93 × 10^−90^	9.61 × 10^−39^	1.01 × 10^−13^	3.61 × 10^−4^	1.69 × 10^−19^	1.13 × 10^−5^
	SD	1.52 × 10^−89^	5.05 × 10^−38^	1.01 × 10^−13^	7.67 × 10^−4^	2.35 × 10^−19^	1.28 × 10^−5^
	*p*	NA	1.73 × 10^−6^	1.73 × 10^−6^	1.73 × 10^−6^	1.73 × 10^−6^	1.73 × 106^5^
	Fri	1	2	4	6	3	5
$F_{5}$	Mean	1.49 × 10^−4^	3.18 × 10^−4^	8.12 × 10^−4^	3.03 × 10^−2^	5.93 × 10^−4^	2.70 × 10^−3^
	SD	1.18 × 10^−4^	2.24 × 10^−4^	5.11 × 10^−4^	1.77 × 10^−2^	4.45 × 10^−4^	1.82 × 10^−3^
	*p*	NA	5.29 × 10^−4^	1.73 × 10^−6^	1.73 × 10^−6^	1.24 × 10^−5^	1.73 × 10^−6^
	Fri	1.3	2.47	3.67	6	2.8	4.77
$F_{6}$	Mean	5.46 × 10^−283^	6.81 × 10^−114^	1.34 × 10^−61^	2.42 × 10^−7^	1.28 × 10^−120^	6.09 × 10^−15^
	SD	0	3.47 × 10^−113^	3.21 × 10^−61^	1.59 × 10^−7^	4.39 × 10^−120^	7.11 × 10^−5^
	*p*	NA	1.73 × 10^−6^	1.73 × 10^−6^	1.73 × 10^−6^	1.73 × 10^−6^	1.73 × 10^−6^
	Fri	1	2	4	6	2	5
$F_{7}$	Mean	1.89 × 10^−182^	1.34 × 10^−89^	2.65 × 10^−31^	1.24 × 10^−7^	6.76 × 10^−58^	2.74 × 10^−12^
	SD	0	7.35 × 10^−89^	4.24 × 10^−31^	1.68 × 10^−7^	2.69 × 10^−57^	3.33 × 10^−12^
	*p*	NA	1.73 × 10^−6^	1.73 × 10^−6^	1.73 × 10^−6^	1.73 × 10^−6^	1.73 × 10^−6^
	Fri	1	2	4	6	3	5
$F_{8}$	Mean	5.87 × 10^−184^	1.15 × 10^−75^	4.82 × 10^−31^	1.29 × 10^−10^	2.18 × 10^−58^	5.87 × 10^−13^
	SD	0	6.29 × 10^−75^	1.09 × 10^−30^	4.82 × 10^−11^	5.98 × 10^−58^	6.02 × 10^−13^
	*p*	NA	1.73 × 10^−6^	1.73 × 10^−6^	1.73 × 10^−6^	1.73 × 10^−6^	1.73 × 10^−6^
	Fri	1	2	4	6	3	5
$F_{9}$	Mean	1.73 × 10^−4^	2.39 × 10^−4^	7.24 × 10^−4^	1.46 × 10^−2^	7.74 × 10^−4^	1.14 × 10^−2^
	SD	1.75 × 10^−4^	2.33 × 10^−4^	4.45 × 10^−4^	8.75 × 10^−3^	4.21 × 10^−4^	6.72 × 10^−3^
	*p*	NA	1.47 × 10^−1^	2.59 × 10^−5^	1.73 × 10^−6^	3.88 × 10^−6^	1.73 × 10^−6^
	Fri	1.67	1.7	3.2	5.63	3.43	5.37
$F_{10}$	Mean	0	0	2.66 × 10^−13^	23.55	8.16 × 10^−1^	2.65
	SD	0	0	1.12 × 10^−12^	10.08	1.91	2.78
	*p*	NA	1	0.5	1.73 × 10^−6^	1.56 × 10^−2^	1.73 × 10^−6^
	Fri	2.35	2.35	2.48	6	2.98	4.83
$F_{11}$	Mean	0	0	0.60	27.33	2.17	1.23
	SD	0	0	1.01	11.85	2.52	1.43
	*p*	NA	1	8.29 × 10^−6^	1.73 × 10^−6^	2.93 × 10^−4^	1.44 × 10^−6^
	Fri	1.78	1.79	3.78	6	3.55	4.1
$F_{12}$	Mean	4.44 × 10^−16^	4.44 × 10^−16^	1.57 × 10^−14^	4.01 × 10^−1^	2.69	11.32
	SD	0	0	1.63 × 10^−14^	1.12	6.98	10.06
	*p*	NA	1	1.49 × 10^−6^	8.29 × 10^−6^	5.08 × 10^−7^	1.73 × 10^−6^
	Fri	1.57	1.57	3.62	4.83	3.92	5.5
$F_{13}$	Mean	0	0	1.73 × 10^−11^	2.14 × 10^−1^	3.08 × 10^−2^	1.02 × 10^−1^
	SD	0	0	8.54 × 10^−11^	1.35 × 10^−1^	4.15 × 10^−2^	9.43 × 10^−2^
	*p*	NA	1	0.13	1.73 × 10^−6^	2.74 × 10^−5^	1.73 × 10^−6^
	Fri	2.05	2.05	2.25	5.87	3.82	4.97
$F_{14}$	Mean	2.59 × 10^−95^	2.90 × 10^−39^	7.22 × 10^−8^	4.53 × 10^−1^	1.19 × 10^−4^	1.26 × 10^−1^
	SD	1.41 × 10^−94^	1.16 × 10^−38^	3.95 × 10^−7^	5.77 × 10^−1^	2.38 × 10^−4^	2.56 × 10^−1^
	*p*	NA	1.73 × 10^−6^	1.73 × 10^−6^	1.73 × 10^−6^	1.73 × 10^−6^	1.73 × 10^−6^
	Fri	1	2	3.4	5.8	3.9	4.9
$F_{15}$	Mean	0	0	0	0	1.74	3.88 × 10^−1^
	SD	0	0	0	0	1.12	2.89
	*p*	NA	1	1	1	3.79 × 10^−6^	2.56 × 10^−6^
	Fri	2.55	2.55	2.55	2.55	5.73	5.07
$F_{16}$	Mean	1.37 × 10^−177^	1.63 × 10^−54^	9.95 × 10^−2^	6.79 × 10^−1^	9.95 × 10^−2^	1.39 × 10^−1^
	SD	0	8.9 × 10^−54^	5.53 × 10^−17^	2.51 × 10^−1^	3.77 × 10^−10^	1.03 × 10^−1^
	*p*	NA	1.73 × 10^−6^	6.79 × 10^−8^	1.25 × 10^−6^	1.73 × 10^−6^	1.72 × 10^−6^
	Fri	1	2	3.07	5.93	4.87	4.13
$F_{17}$	Mean	0	0	0	2.15	0	2.12 × 10^−13^
	SD	0	0	0	1.19	0	2.32 × 10^−13^
	*p*	NA	1	1	1.73 × 10^−6^	1.73 × 10^−6^	1.73 × 10^−6^
	Fri	2.5	2.5	2.5	6	2.5	5
$F_{18}$	Mean	3.99 × 10^−1^	3.98 × 10^−1^	3.98 × 10^−1^	3.98 × 10^−1^	3.98 × 10^−1^	3.98 × 10^−1^
	SD	2.83 × 10^−3^	0	9.75 × 10^−15^	1.04 × 10^−13^	6.76 × 10^−6^	3.22 × 10^−11^
	*p*	NA	1.73 × 10^−6^	1.73 × 10^−6^	1.73 × 10^−6^	1.73 × 10^−6^	1.73 × 10^−6^
	Fri	6	1.35	1.72	2.93	5	4

Note: NA in the table indicates invalid data.

**Table 6:** Simulation results of MBKA and BKA on CEC-2017.

Function	MBKA				BKA				
	Max	Mean	Min	SD	Max	Mean	Min	SD	*p*-Values
${CEC2017-F}_{1}$	2.13 × 10^9^	1.05 × 10^9^	6.44 × 10^7^	5.59 × 10^8^	2.15 × 10^9^	4.27 × 10^7^	1.01 × 10^7^	5.25 × 10^8^	2.41 × 10^−4^
${CEC2017-F}_{3}$	5.19 × 10^3^	2.92 × 10^3^	8.73 × 10^2^	1.19 × 10^3^	9.71 × 10^3^	3.14 × 10^3^	7.72 × 10^2^	1.91 × 10^3^	8.93 × 10^−1^
$CEC2017{-F}_{4}$	5.4 × 10^2^	4.79 × 10^2^	4.31 × 10^2^	2.49 × 10^1^	8.75 × 10^2^	5.42 × 10^2^	4.14 × 10^2^	9.82 × 10^1^	5.67 × 10^−3^
${CEC2017-F}_{5}$	5.68 × 10^2^	5.53 × 10^2^	5.28 × 10^2^	8.29	5.92 × 10^2^	5.59 × 10^2^	5.33 × 10^2^	1.8 × 10^1^	2.8 × 10^−1^
${CEC2017-F}_{6}$	6.37 × 10^2^	6.23 × 10^2^	6.12 × 10^2^	6.33	6.45 × 10^2^	6.31 × 10^2^	6.16 × 10^2^	7.29	6.16 × 10^−4^
$CEC2017-F_{7}$	7.85 × 10^2^	7.71 × 10^2^	7.59 × 10^2^	7.92	8.09 × 10^2^	7.72 × 10^2^	7.33 × 10^2^	21.75	8.94 × 10^1^
$CEC2017-F_{8}$	8.56 × 10^2^	8.43 × 10^2^	8.19 × 10^2^	8.38	8.5 × 10^2^	8.31 × 10^2^	8.13 × 10^2^	9.49	1.36 × 10^4^
$CEC2017-F_{9}$	1.28 × 10^3^	1.06 × 10^3^	9.32 × 10^2^	8.41 × 10^1^	1.77 × 10^3^	1.28 × 10^3^	9.53 × 10^2^	1.88 × 10^2^	3.41 × 10^−2^
$CEC2017{-F}_{10}$	2.49 × 10^3^	2.23 × 10^3^	1.74 × 10^3^	1.56 × 10^2^	3.01 × 10^3^	2.3 × 10^3^	1.44 × 10^3^	3.76 × 10^2^	3.82 × 10^−1^
$CEC2017{-F}_{11}$	2.14 × 10^3^	1.62 × 10^3^	1.23 × 10^3^	2.17 × 10^2^	1.49 × 10^3^	1.26 × 10^3^	1.13 × 10^3^	1.04 × 10^2^	3.88 × 10^−6^
${CEC2017-F}_{12}$	1.03 × 10^8^	2.89 × 10^7^	1.52 × 10^6^	2.84 × 10^7^	1.22 × 10^7^	2.49 × 10^6^	1.71 × 10^4^	3.42 × 10^6^	3.52 × 10^−2^
${CEC2017-F}_{13}$	1.67 × 10^5^	5.51 × 10^4^	7.01 × 10^3^	4.43 × 10^4^	1.74 × 10^4^	4.82 × 10^3^	1.56 × 10^2^	4.26 × 10^3^	1.92 × 10^−6^
$CEC2017-F_{14}$	2.83 × 10^3^	1.85 × 10^3^	1.53 × 10^3^	2.64 × 10^2^	1.59 × 10^3^	1.5 × 10^3^	1.44 × 10^3^	4.18 × 10^2^	1.73 × 10^−6^
${CEC2017-F}_{15}$	1.28 × 10^4^	5.98 × 10^3^	2.58 × 10^3^	2.61 × 10^3^	7.23 × 10^3^	2 × 10^3^	1.53 × 10^3^	1.02 × 10^3^	7.69 × 10^−6^
$CEC2017-F_{16}$	1.99 × 10^3^	1.83 × 10^3^	1.65 × 10^3^	8.13 × 10^1^	2.07 × 10^3^	1.88 × 10^3^	1.72 × 10^3^	8.84 × 10^1^	1.32 × 10^2^
$CEC2017-F_{17}$	1.81 × 10^3^	1.45 × 10^5^	1.76 × 10^3^	1.17 × 10^1^	1.94 × 10^3^	1.79 × 10^3^	1.74 × 10^3^	4.37 × 10^1^	7.19 × 10^−1^
${CEC2017-F}_{18}$	5.34 × 10^5^	3.24 × 10^3^	2.28 × 10^4^	1.39 × 10^5^	2.56 × 10^4^	5.88 × 10^3^	1.97 × 10^3^	4.55 × 10^3^	1.73 × 10^−6^
${CEC2017-F}_{19}$	1.18 × 10^5^	2.11 × 10^4^	2.83 × 10^3^	2.32 × 10^4^	7.29 × 10^3^	2.34 × 10^3^	1.92 × 10^3^	1.05 × 10^3^	1.73 × 10^−6^
${CEC2017-F}_{20}$	2.24 × 10^3^	2.15 × 10^3^	2.07 × 10^3^	4.26 × 10^1^	2.26 × 10^3^	2.16 × 10^3^	2.08 × 10^3^	5.51 × 10^1^	6.88 × 10^−1^
$CEC2017-F_{21}$	2.35 × 10^3^	2.25 × 10^3^	2.22 × 10^3^	3.45 × 10^1^	2.38 × 10^3^	2.29 × 10^3^	2.2 × 10^3^	6.56 × 10^1^	1.11 × 10^−2^
${CEC2017-F}_{22}$	2.52 × 10^3^	2.44 × 10^3^	2.28 × 10^3^	4.27 × 10^1^	3.45 × 10^3^	2.48 × 10^3^	2.29 × 10^3^	1.74 × 10^2^	2.06 × 10^−1^
${CEC2017-F}_{23}$	2.67 × 10^3^	2.65 × 10^3^	2.63 × 10^3^	8.35	2.73 × 10^3^	2.65 × 10^3^	2.62 × 10^3^	2.92 × 10^1^	9.59 × 10^−1^
${CEC2017-F}_{24}$	2.79 × 10^3^	2.77 × 10^3^	2.59 × 10^3^	4.25 × 10^1^	2.84 × 10^3^	2.78 × 10^3^	2.54 × 10^3^	6.12 × 10^1^	1.85 × 10^−1^
$CEC2017-F_{25}$	3.11 × 10^3^	3.01 × 10^3^	2.95 × 10^3^	4.21 × 10^1^	3.25 × 10^3^	3.04 × 10^3^	2.92 × 10^3^	8.34 × 10^1^	2.45 × 10^−1^
$CEC2017-F_{26}$	3.4 × 10^3^	3.16 × 10^3^	3.04 × 10^3^	8.83 × 10^1^	4.55 × 10^3^	3.56 × 10^3^	2.75 × 10^3^	4.87 × 10^2^	2.41 × 10^−4^
$CEC2017{-F}_{27}$	3.11 × 10^3^	3.11 × 10^3^	3.1 × 10^3^	1.76	3.31 × 10^3^	3.14 × 10^3^	3.09 × 10^3^	4.34 × 10^1^	1.49 × 10^−5^
$CEC2017-F_{28}$	3.42 × 10^3^	3.31 × 10^3^	3.23 × 10^3^	5.21 × 10^1^	3.79 × 10^3^	3.42 × 10^3^	3.18 × 10^3^	1.71 × 10^2^	6.04 × 10^−3^
${CEC2017-F}_{29}$	3.33 × 10^3^	3.24 × 10^3^	3.17 × 10^3^	4.05 × 10^1^	3.44 × 10^3^	3.29 × 10^3^	3.17 × 10^3^	5.81 × 10^1^	4.89 × 10^−4^
${CEC2017-F}_{30}$	8.86 × 10^5^	8.37 × 10^5^	4.72 × 10^4^	1.64 × 10^5^	1.88 × 10^7^	3 × 10^6^	4.16 × 10^3^	4.07 × 10^6^	5.47 × 10^−3^

**Table 7:** Simulation results of MBKA and BKA on CEC-2022.

Function	MBKA				BKA				
	Max	Mean	Min	SD	Max	Mean	Min	SD	*p*-Values
${CEC2022-F}_{1}$	30,001.25	13,103.73	7063.04	4069.28	7625.07	3190.79	573.08	1865.35	1.73 × 10^−6^
$CEC2022-F_{2}$	580.19	498.35	464.34	23.56	811.28	534.48	422.29	109.14	3.82 × 10^−1^
${CEC2022-F}_{3}$	635.67	623.88	608.88	5.72	654.09	633.69	615.77	10.78	3.07 × 10^−4^
${CEC2022-F}_{4}$	852.79	839.36	822.40	5.97	850.10	831.89	815.09	7.70	3.24 × 10^−5^
$CEC2022{-F}_{5}$	160.79	1031.04	937.67	50.66	1633.73	1197.97	939.72	160.79	5.31 × 10^−5^
${CEC2022-F}_{6}$	23.52 × 10^6^	9.54 × 10^5^	1.34 × 10^5^	5.09 × 10^6^	8234.00	3342.08	1884.13	1777.41	7.57 × 10^−10^
$CEC2022-F_{7}$	2086.97	2061.5	2044.87	9.75	2103.23	2064.31	2027.78	20.29	5.04 × 10^−1^
$CEC2022-F_{8}$	2239.44	2233.49	2226.33	3.39	2354.19	2235.17	2222.92	24.22	1.25 × 10^−2^
$CEC2022-F_{9}$	2720.43	2636.31	2576.15	33.05	2727.31	2642.85	2540.98	50.17	4.53 × 10^−1^
${CEC2022-F}_{10}$	2642.09	2505.59	2500.89	25.23	3053.04	2563.00	2500.64	114.74	1.66 × 10^−2^
$CEC2022{-F}_{11}$	3420.09	2845.97	2769.09	121.70	4069.56	3156.64	2770.26	381.55	2.61 × 10^−4^
$CEC2022{-F}_{12}$	2873.43	2871.17	2867.99	1.46	2973.33	2902.04	2868.23	30.22	6.98 × 10^−6^

**Table 8:** Compression spring design problem’s optimal outcomes from various algorithms.

Algorithms	Optimal Values for Variables			Best	Avg.	Std.
	d	D	N			
MBKA	5.43 × 10^−2^	0.4220	8.2968	1.28 × 10^−2^	1.978 × 10^−2^	1.6658 × 10^−4^
BKA	5.91 × 10^−2^	0.5633	4.9074	1.36 × 10^−2^	1.3329 × 10^−2^	9.2757 × 10^−4^
WOA	5.85 × 10^−2^	0.5463	5.2335	1.35 × 10^−2^	1.3917 × 10^−2^	1.5035 × 10^−3^
ALO	5.70 × 10^−2^	0.4995	6.0958	1.32 × 10^−2^	1.36 × 10^−2^	1.421 × 10^−3^
DO	5.51 × 10^−2^	0.4447	7.5292	1.29	1.4021 × 10^−2^	1.2076 × 10^−3^

**Table 9:** Gear train design problem’s optimal outcomes from various algorithms.

Algorithms	Optimal Values for Variables				Best	Avg.	Std.
	$n_{A}$	$n_{B}$	$n_{C}$	$n_{D}$			
MBKA	51.439	26.283	14.938	52.745	2.3078 × 10^−11^	1.4425 × 10^−9^	1.649 × 10^−9^
BKA	47.367	12.558	12.485	22.823	9.9216 × 10^−10^	1.8605 × 10^−9^	3.7157 × 10^−9^
WOA	46.758	12.193	12.519	23.109	9.9216 × 10^−10^	3.7376 × 10^−9^	6.3414 × 10^−9^
ALO	42.759	12.000	14.563	29.113	4.5033 × 10^−9^	6.8496 × 10^−9^	7.7234 × 10^−9^
DO	55.567	13.366	22.582	37.081	6.6021 × 10^−10^	1.6529 × 10^−9^	1.2562 × 10^−9^

## Data Availability

The data that support the findings of this study are available from the corresponding author upon request. There are no restrictions on data availability.
